# Symmetric inheritance of parental histones governs epigenome maintenance and embryonic stem cell identity

**DOI:** 10.1038/s41588-023-01476-x

**Published:** 2023-09-04

**Authors:** Alice Wenger, Alva Biran, Nicolas Alcaraz, Alba Redó-Riveiro, Annika Charlotte Sell, Robert Krautz, Valentin Flury, Nazaret Reverón-Gómez, Victor Solis-Mezarino, Moritz Völker-Albert, Axel Imhof, Robin Andersson, Joshua M. Brickman, Anja Groth

**Affiliations:** 1 Novo Nordisk Foundation Center for Protein Research (CPR), University of Copenhagen, Copenhagen, Denmark; 2 Biotech Research and Innovation Centre (BRIC), University of Copenhagen, Copenhagen, Denmark; 3 Section for Computational and RNA Biology, Department of Biology, University of Copenhagen, Copenhagen, Denmark; 4 Novo Nordisk Foundation Center for Stem Cell Medicine (reNEW), University of Copenhagen, Copenhagen, Denmark; 5 EpiQMAx GmbH, Planegg, Germany; 6 Faculty of Medicine, Biomedical Center, Protein Analysis Unit, Ludwig-Maximilians-Universität München, Planegg, Germany; 7 The Novo Nordisk Foundation Center for Genomic Mechanisms of Disease, Broad Institute of MIT and Harvard, Cambridge, MA USA; 8 Department of Cellular and Molecular Medicine (ICMM), University of Copenhagen, Copenhagen, Denmark; 9 Present Address: Lexogen GmbH, Vienna, Austria

**Keywords:** Epigenetics, Stem cells, Epigenomics

## Abstract

Modified parental histones are segregated symmetrically to daughter DNA strands during replication and can be inherited through mitosis. How this may sustain the epigenome and cell identity remains unknown. Here we show that transmission of histone-based information during DNA replication maintains epigenome fidelity and embryonic stem cell plasticity. Asymmetric segregation of parental histones H3–H4 in MCM2-2A mutants compromised mitotic inheritance of histone modifications and globally altered the epigenome. This included widespread spurious deposition of repressive modifications, suggesting elevated epigenetic noise. Moreover, H3K9me3 loss at repeats caused derepression and H3K27me3 redistribution across bivalent promoters correlated with misexpression of developmental genes. MCM2-2A mutation challenged dynamic transitions in cellular states across the cell cycle, enhancing naïve pluripotency and reducing lineage priming in G1. Furthermore, developmental competence was diminished, correlating with impaired exit from pluripotency. Collectively, this argues that epigenetic inheritance of histone modifications maintains a correctly balanced and dynamic chromatin landscape able to support mammalian cell differentiation.

## Abstract (web summary)

Asymmetric segregation of parental histones H3 and H4 in MCM2-mutant embryonic stem cells impacts mitotic inheritance of histone modifications and genome regulation. MCM2-2A mutation perturbs exit from pluripotency and differentiation.

## Main

During development, cellular specification is established gradually in the backdrop of multiple mitotic cell divisions. This process requires the inactivation of early developmental cell states in favor of progressive activation of defined lineage-specific cell types, orchestrated by extracellular signaling, transcription factors and chromatin regulators^1–3^. Notably, cell-fate trajectories and cellular identity can be maintained across generations, in part through epigenetic regulation^3,4^. Histone post-translational modifications (PTMs) are attractive mediators of epigenetic cell memory^5–8^, but the role of histone-based inheritance in governing the identity of mammalian cells remains a longstanding question.

**Fig. 1:**
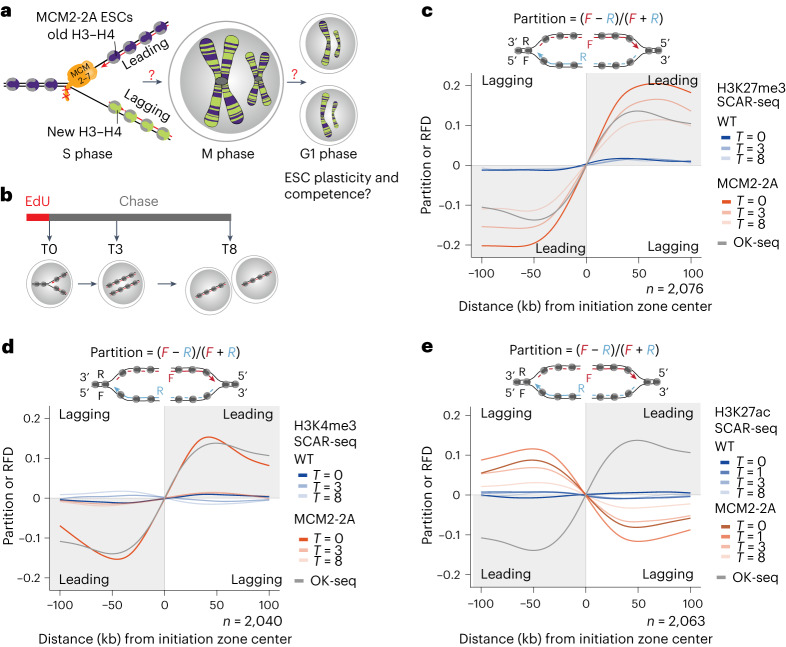
Symmetric segregation of parental histones is required for the balanced inheritance of histone PTMs to daughter cells. **a**, Illustration of asymmetric segregation of parental histones H3–H4 to leading strand in MCM2-2A ESCs^14^ and how this could challenge histone PTM inheritance and daughter cell function. **b**, Design of SCAR-seq pulse-chase experiments. **c**–**e**, Average SCAR-seq profiles of H3K27me3 (**c**), H3K4me3 (**d**) and H3K27ac (**e**) partition in 1-kb windows around replication initiation zones. Partition is calculated as the proportion of forward (*F*) and reverse (*R*) read counts ((*F* − *R*)/(*F* + *R*))^14^. *n* = number of initiation zones. Replication fork directionality in WT cells^14^ measured by Okazaki fragment sequencing (OK-seq)^85^ is shown for comparison. The average of two biological replicates is shown (see Extended Data Figs. 2 and 3 for individual replicates in two MCM2-2A clones).

DNA replication disrupts chromatin on the parental DNA strand, and nucleosomes are rapidly reassembled on daughter DNA strands from old parental histones and new largely unmodified histones^8,9^. Parental histones H3–H4 are recycled with their PTMs to the two daughter DNA strands, accurately^10–12^ and largely symmetrically^13–15^, by the DNA replication machinery. MCM2, part of the CDC45-MCM2-7-GINS (CMG) helicase, and DNA polymerase α recycle parental histones H3–H4 to the lagging strand^13,14,16^, and DNA polymerase epsilon subunits POLE3/POLE4 promote recycling to the leading strand^15,16^. Following deposition, new naïve histones are modified with PTM- and locus-specific kinetics to restore pre-replication PTM levels^10,17^ in a manner thought to be stimulated by modifications on recycled parental histones^7–9,18^. Whereas H3K4me3, associated with active transcription, is rapidly restored to pre-replication levels, restoration of the repressive modifications H3K27me3 and H3K9me3 is slow and continues after mitosis in daughter cells^10,17,19^. Positive feedback can contribute to the propagation of H3K27me3 and H3K9me3 through read–write mechanisms^6,7,19–25^, where modified parental histones stimulate similar modification of neighboring new histones, for example, by allosteric activation of PRC2 and SUV39h1/SUV39h2 (refs. ^22,24,26^). In addition, crosstalk between modifications^18^ both on H3–H4 and H2A–H2B^27^ might also contribute with both negative and positive feedback to post-replication chromatin restoration. Recycling of parental histones to both daughter strands is thus predicted to underlie the propagation of histone PTMs to daughter cells, which in turn is thought to maintain daughter cell identity^3,5,6,8,9^. Asymmetric recycling of parental histones impairs the silencing of some repetitive regions in yeast^28–30^ and mouse embryonic stem cells (ESCs)^16^. Furthermore, asymmetric recycling of parental histones may underlie the unbalanced transmission of new and old histones to daughter cells during asymmetric divisions of *Drosophila* germline stem cells^31,32^, which is proposed to guide distinct cell-fate trajectories. However, the significance of symmetric histone segregation for epigenome inheritance remains unclear and the epigenetic function of histone PTMs in shaping cell identity is debated. Here we explore the consequences of asymmetric parental histone segregation during DNA replication for histone PTM inheritance and maintenance, genome regulation and pluripotent cell identity. We use mouse ESCs carrying two mutations in the MCM2 histone-binding domain (MCM2-2A), which cause asymmetric parental histone recycling to the leading strand without affecting DNA replication^14^ (Fig. 1a).

## Results

### Imbalanced inheritance of histone PTMs in MCM2-2A mutants

**Extended Data Fig. 1:**
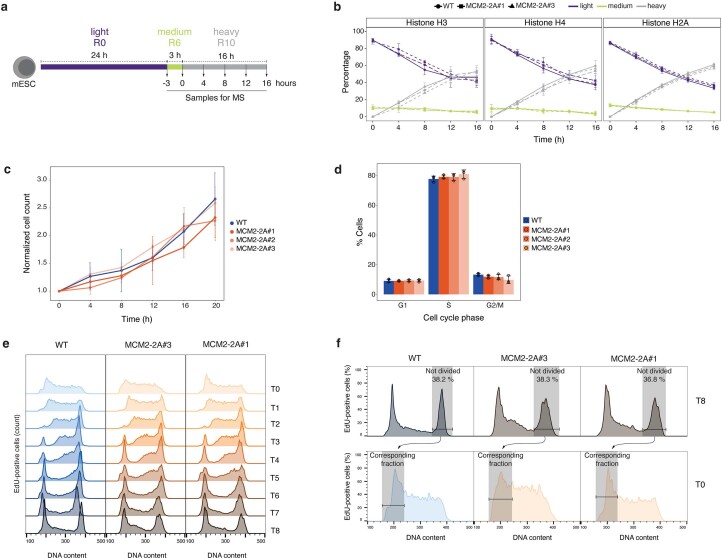
Histone dynamics and cell cycle progression in MCM2-2A mESCs. **a**, Experimental setup for pulse-SILAC MS analysis of new histone incorporation and old histone dilution kinetics. **b**, Relative levels of old histones (purple), new pulse-labeled histones (green) and new steady-state labeled (gray) histones over time. Mean ± s.d. of *n* = 3 biological replicates is shown. **c**, Growth curves of WT and three MCM2-2A mESC clones. Cell counts are shown relative to the first time point. Mean ± s.d. of *n* = 3 biological replicates is shown. **d**, Cell-cycle analysis of WT and three MCM2-2A clones. The fraction of cells in G1-, S- and G2/M-phase was determined by flow cytometry analysis of DNA content and EdU labeling. Mean ± s.d. of *n* = 3 biological replicates is shown, dots depict individual data points. **e**, Flow cytometry analysis showing that cell cycle progression of EdU-labeled cells is similar in WT and two MCM2-2A clones. Cells were pulsed with EdU for 15 min and harvested immediately (T0) or at the indicated time points (T1-T8). Note that most labeled cells have divided at the 8-hour time point. Data are representative of *n* = 2 biological replicates. **f**, Quantification of EdU-labeled cells which have not divided at the 8-hour timepoint in **e**. This represents the cells labeled by EdU in early S. Source data

**Extended Data Fig. 2:**
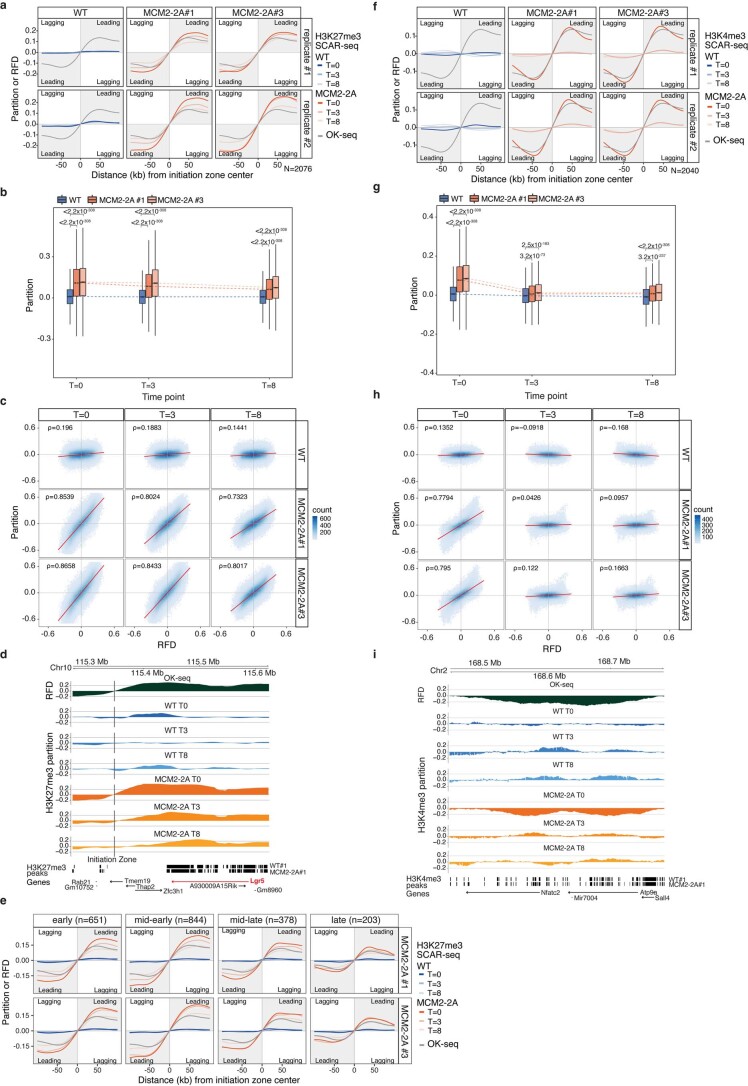
Histone H3K27me3 asymmetry is inherited by MCM2-2A daughter cells, but H3K4me3 asymmetry is rapidly resolved. **a**, Individual biological replicates of H3K27me3 SCAR-seq profiles related to Fig. 1c, including two MCM2-2A clones. SCAR-seq is represented as in Fig. 1. **b**, Box plots of H3K27me3 partition in 1 kb windows around the RFD extrema (distance of 10-90 kb from initiation zones). Windows upstream of initiation zones were multiplied by -1. Box plots as in Fig. 2a. Dashed lines illustrate trends of partition changes over time. Significance was tested per time point and replicate between WT and MCM2-2A using two-sided paired Wilcoxon signed-rank test. **c**, Scatter plots of H3K27me3 SCAR-seq partition and RFD show association between histone segregation and replication fork directionality. Two-sided Spearman’s rank correlation coefficient. **d**, Example region of H3K27me3 SCAR-seq in WT and MCM2-2A#1 and RFD^14^ containing a differentially expressed gene (Lgr5; Fig. 4d, Extended Data Fig. 7g). Chr10:115272208-115624981. **e**, H3K27me3 SCAR-seq profiles separated according to replication timing, showing that asymmetry at T8 is independent of replication timing. As all cells labeled in mid and late S phase have divided at T8 (Extended Data Fig. 1f), asymmetry is transmitted to daughter cells. Average profiles of RFD and H3K27me3 are represented as in Fig. 1. RFD amplitudes and thus also SCAR-seq amplitudes are lower around late compared to early initiation zones due to more heterogenous replication fork progression across the cell population^85^, but the relative differences between the amplitudes are similar across the replication timing categories. **b**–**e**, The average of *n* = 2 biological replicates is shown for each clone. **f**, Individual biological replicates of H3K4me3 SCAR-seq related to Fig. 1d, including two MCM2-2A clones. SCAR-seq is shown as in Fig. 1. **g**, Box plots of H3K4me3 partition in 1 kb windows around the RFD extrema (distance of 10-90 kb from initiation zones) as in **b**. **h**, Scatter plots of H3K4me3 SCAR-seq partition and RFD show association between histone segregation and replication fork directionality as in **c**. **i**, Example region of H3K4me3 SCAR-seq in WT and MCM2-2A#1 and RFD^14^ containing a highly expressed pluripotency gene (Sall4). Chr2:168450809-168783596. **g-i**, The average of *n* = 2 biological replicates is shown.

In MCM2-2A ESCs, parental histone PTMs are strongly enriched on the leading strand shortly after DNA replication, creating alternating patterns of parental and new histones along each sister chromatid^14^ (Fig. 1a). Parental histones are not lost at replication forks but rerouted from the lagging to the leading strand, as the gradual dilution of old histones and the incorporation dynamics of new histones across the cell cycle are unaltered (Extended Data Fig. 1a,b). In addition, MCM2-2A mutants showed similar growth rates and cell cycle distribution as wild-type (WT) cells (Extended Data Fig. 1c,d)^14^. To address the effect of asymmetric recycling on histone PTM inheritance during mitosis, we followed sister chromatid asymmetry post-replication and into daughter cells by sister chromatids after replication (SCAR)-seq. In SCAR-seq, replicating DNA is labeled with a short 5-ethynyl-2′-deoxyuridine (EdU) pulse before sequential native chromatin immunoprecipitation (ChIP) and EdU pull-down followed by strand-specific sequencing^14^. We performed pulse-chase SCAR-seq of H3K27me3, H3K4me3 and H3K27ac to track asymmetry on nascent chromatin (0 h), in late S phase/G2 phase (3 h) and after mitosis in daughter cells (8 h; Fig. 1b and Extended Data Fig. 1e,f). As expected for modifications on parental histones^14,16^, H3K27me3 showed a strong leading strand bias in nascent chromatin of MCM2-2A cells (Fig. 1c and Extended Data Fig. 2a–d). This asymmetry decreased over time, demonstrating that the establishment of H3K27me3 takes place on the lagging strand despite a strongly reduced contribution of neighboring parental histones. However, substantial H3K27me3 asymmetry was present 8 h post-replication in daughter cells (Fig. 1c and Extended Data Fig. 2a–e), indicating that de novo establishment fails to compensate for the strong asymmetry generated during replication. This is likely explained by reduced allosteric PRC2 activation on the lagging strand along with the generally slow kinetics of H3K27me3 establishment on new histones^17,19^.

H3K4me3 was also segregated asymmetrically to the leading strand in MCM2-2A cells (Fig. 1d and Extended Data Fig. 2f–i), demonstrating that parental histones in active chromatin are also recycled during DNA replication. In contrast to H3K27me3, balanced H3K4me3 occupancy was restored within 3 h and thus before mitosis. This is consistent with the rapid restoration kinetics of H3K4me3 (ref. ^10^) associated with transcription restart^33^ and suggests that an epigenetic contribution from recycled parental histones is not critical to restore H3K4me3 levels.

**Extended Data Fig. 3:**
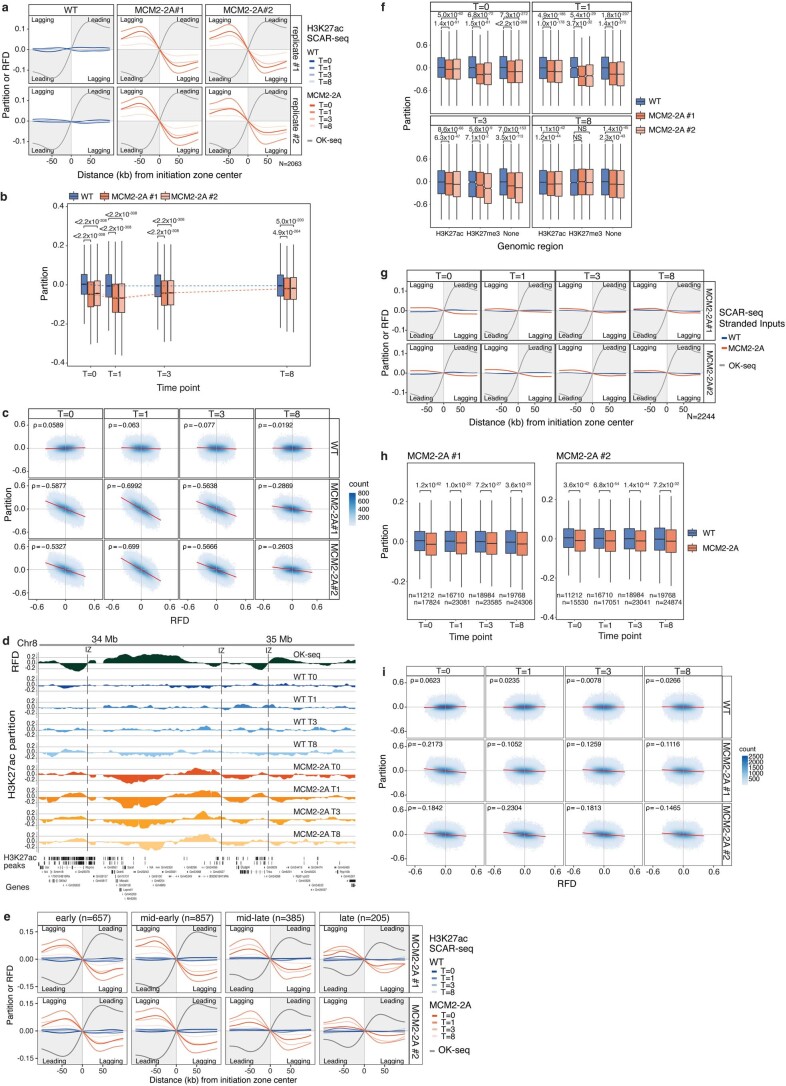
Persistent genome-wide H3K27ac asymmetry biased towards the lagging strand in MCM2-2A cells. **a**, Individual biological replicates of H3K27ac SCAR-seq related to Fig. 1e, including two MCM2-2A clones. SCAR-seq is represented as in Fig. 1. **b**, Box plots of H3K27ac partition in 1 kb windows around the RFD extrema (distance of 10–90 kb from initiation zones) as in Extended Data Fig. 2b. **c**, Scatter plots of H3K27ac SCAR-seq partition and RFD showing negative correlation between histone H3K27ac and replication fork directionality, demonstrating a lagging strand bias. Two-sided Spearman’s rank correlation coefficient. **d**, Example region of H3K27ac SCAR-seq in WT and MCM2-2A#1 clones and RFD^14^ containing genic and non-genic regions. Chr8:33670000-35487509. **e**, H3K27ac SCAR-seq profiles separated according to replication timing (as in Extended Data Fig. 2e) showing H3K27ac asymmetry at T8 at early, mid-early, mid-late and late replicating regions. **f**, H3K27ac asymmetry is present genome-wide in MCM2-2A cells. Box plots of H3K27ac SCAR-seq in 1 kb windows overlapping H3K27ac and H3K27me3 peaks or neither of the two. Box plots as in Fig. 2a. **g**, Slight increase in lagging strand accessibility in MCM2-2A cells, indicated by lagging strand bias of MNase inputs. Average profiles of stranded inputs in 1 kb windows around replication initiation zones. Partition is calculated as the proportion of forward (F) and reverse (R) read counts. RFD in WT cells^14^ measured by Okazaki fragment sequencing (OK-seq) is shown for comparison. **h**, Box plot of stranded input profiles shown in (**g**) in 1 kb windows around the RFD extrema (distance of 15–75 kb from initiation zones). Box plots as in Fig. 2a. **i**, Scatter plots of stranded inputs and RFD showing negative correlation between MNase accessibility and replication fork directionality in MCM2-2A cells, demonstrating a slight lagging strand bias. Two-sided Spearman’s rank correlation coefficient is shown in the top left corner. **b-i**, The average of *n* = 2 biological replicates is shown.

In contrast to H3K27me3 and H3K4me3, H3K27ac showed a lagging strand bias in MCM2-2A nascent chromatin similar to new histones^14^ (Fig. 1e and Extended Data Fig. 3a–e). This argues that the majority of H3K27ac in nascent chromatin is found on new histones and not on recycled parental histones. New histones are not modified by H3K27ac pre-deposition^34^, but H3K27ac has been proposed to occur on new histones in nascent chromatin^35^. Consistent with this, the lagging strand bias in nascent chromatin was present genome-wide and not restricted to H3K27ac-marked regions like enhancers (Extended Data Fig. 3f). The degree of H3K27ac asymmetry increased during the first-hour post-replication before resolving gradually (Fig. 1e and Extended Data Fig. 3a–f), reflecting a transient post-replication wave of H3K27ac modifications on new lagging strand histones. MCM2-2A cells showed H3K27ac asymmetry even 8 h post-replication, underscoring that the unbalanced distribution of new and old histones translates into defects in chromatin restoration that propagate to daughter cells. Consistent with distinct chromatin states on the two strands, we observed a small but consistent asymmetry in MNase accessibility biased toward the lagging strand throughout the time course (Extended Data Fig. 3g–i). Collectively, this demonstrates that a balanced contribution of parental and new histones on newly synthesized DNA strands is required for proper chromatin restoration and symmetric inheritance of the histone PTM landscape to daughter cells.

### Asymmetric histone segregation reconfigures the epigenome

**Fig. 2:**
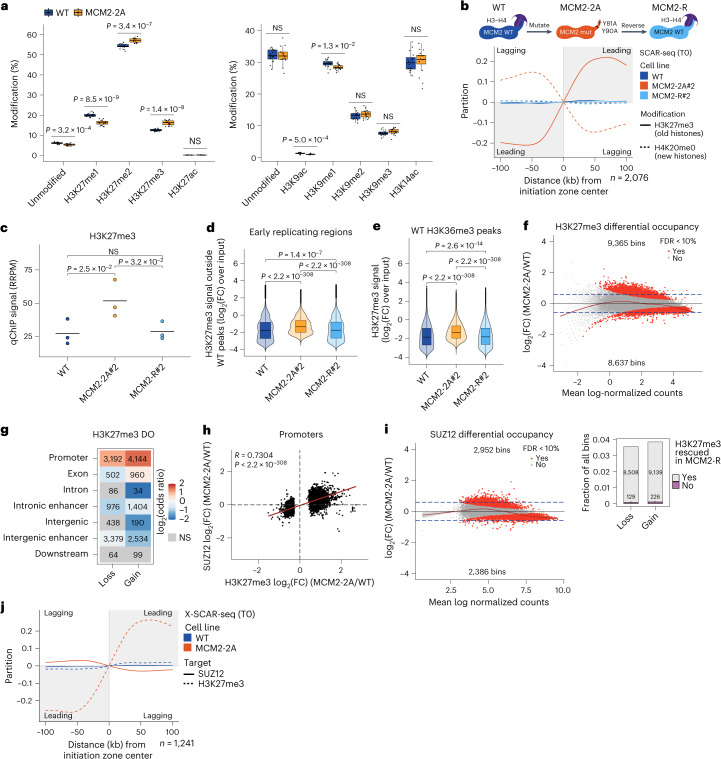
MCM2-2A cells show unscheduled H3K27me3 accumulation. **a**, Global histone PTM levels quantified by mass spectrometry. *n* = biological replicates; WT#1 (*n* = 4), WT#2 (*n* = 4), WT#3 (*n* = 4), WT#4 (*n* = 4), MCM2-2A#1 (*n* = 4), MCM2-2A#3 (*n* = 4), MCM2-2A#4 (*n* = 4) and MCM2-2A#5 (*n* = 4). Two-sided *t* test. Lines indicate median, boxes represent first and third quartiles and whiskers extend 1.5× IQR. **b**, SCAR-seq profiles showing symmetric histone segregation in MCM2-R cells (as in Fig. 1). **c**, Global H3K27me3 levels measured by qChIP–seq. *n* = 3 biological replicates. Two-sided paired *t* test. **d**, H3K27me3 signal in 5-kb bins outside WT peaks in early replicating regions. *n* = 3 biological replicates. Two-sided Wilcoxon signed-rank test. Box plots as in **a**. **e**, H3K27me3 signal overlapping WT H3K36me3 peaks. *n* = 3 biological replicates. Two-sided Wilcoxon signed-rank test. Box plots as in **a**. **f**, H3K27me3 differential occupancy (DO) in MCM2-2A#2 versus WT in 5-kb bins overlapping H3K27me3 WT peaks (top) and bar plot showing rescue in MCM2-R 2 (bottom). Significant DO bins (red), False discovery rate (FDR) < 0.1, Bayes quasi-likelihood *F* test (Supplementary Methods). *n* = 3 biological replicates. **g**, Enrichment analysis (odds ratios) of H3K27me3 DO according to genome annotation. Significant states (*P* < 0.001, two-sided Fisher’s exact test) are colored according to enrichment (red) or depletion (blue), and NS states are shown in gray. *n* = number of bins. **h**, Correlation of H3K27me3 and SUZ12 DO at promoters with DO of H3K27me3 (*n* = 6,660). Two-sided Pearson’s correlation coefficient (*R*) with *P* value. Average of *n* = 3 biological replicates. **i**, SUZ12 DO in MCM2-2A 2 versus WT in 2.5-kb bins overlapping SUZ12 WT and MCM2-2A peaks. *n* = 3 biological replicates. Significant DO bins (red), FDR < 0.1, Bayes quasi-likelihood *F* test. **j**, Average crosslinked SCAR-seq profiles as in Fig. 1. IQR, interquartile range; NS, not significant. Source data

**Extended Data Fig. 4:**
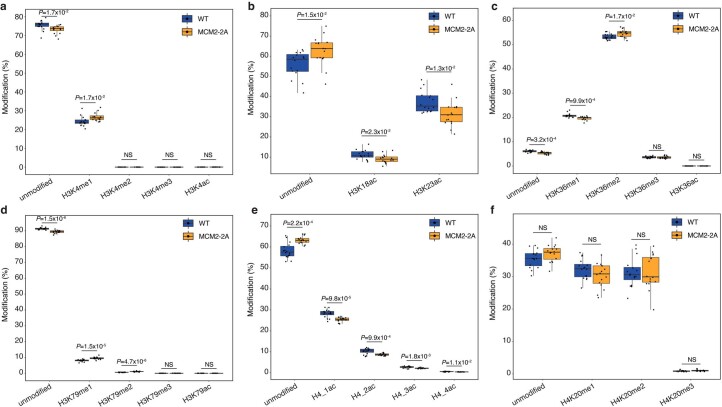
Global levels of H3-H4 modifications. **a-f**, Box plots showing relative levels of H3K4 methylation and acetylation (**a)**, H3K18 and H3K23 acetylation (**b**), H3K36 methylation (**c**), H3K79 methylation and acetylation (**d**), H4K5/8/12/16 acetylation (**e**), and H4K20 methylation (**f**). Histone modifications were quantified by mass spectrometry in WT and MCM2-2A cells growing asynchronously. WT#1 (*n* = 4), WT#2 (*n* = 4), WT#3 (*n* = 4), WT#4 (*n* = 4), MCM2-2A#1 (*n* = 4), MCM2-2A#3 (*n* = 4), MCM2-2A#4 (*n* = 4), MCM2-2A#1 (*n* = 4); *n* = biological replicates. Lines represent median, boxes represent the 1st and 3rd quartiles, whiskers extend 1.5x interquartile range (IQR), dots depict individual data points. Two-sided Welch’s t-test FDR. Source data

**Extended Data Fig. 5:**
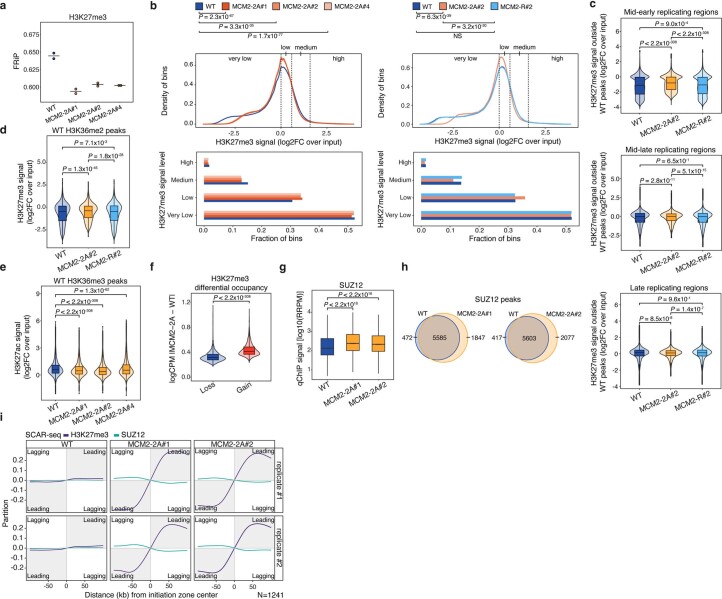
Global increase of H3K27me3 and SUZ12 levels in MCM2-2A. **a**, Fraction of reads in peaks (FRiP) as quality control for H3K27me3 ChIP-seq peaks. Fewer reads fall into peaks in MCM2-2A clones compared to WT cells, indicating that peak calling for H3K27me3 is affected in MCM2-2A (*n* = 2 biological replicates for each clone). **b**, Density plots (top) showing distribution of H3K27me3 signal in genome-wide 5-kb bins. Two-sided Wilcoxon signed-rank test *P* values. Bar plots (bottom) showing quantification of bins in the indicated signal categories. Three MCM2-2A clones (left; average of *n* = 2 biological replicates) and MCM2-R cells (right; average of *n* = 3 biological replicates) demonstrate similar trends across mutant clones and rescue of signal redistribution upon restoration of symmetric recycling. **c**, Violin plots showing H3K27me3 signal in bins non-overlapping WT peaks in early, mid-early, mid-late and late replicating regions, related to Fig. 2d. Two-sided Wilcoxon signed-rank test *P* values. *n* = 3 biological replicates. **d**, Violin plot showing H3K27me3 signal in 5 kb bins overlapping WT H3K36me2 peaks. Two-sided Wilcoxon signed-rank test *P* values. *n* = 3 biological replicates. **e**, Violin plot showing H3K27ac signal in 2.5-kb bins overlapping WT H3K36me3 peaks. Two-sided Wilcoxon signed-rank test *P* values. *n* = 2 biological replicates. **f**, Violin plot showing absolute differences in H3K27me3 signal between WT and MCM2-2A in bins of H3K27me3 gains and H3K27me3 losses. Two-sided Wilcoxon signed-rank test *P* values. *n* = 3 biological replicates. Related to Fig. 2f. **g**, Increased SUZ12 binding to chromatin in MCM2-2A cells shown by box plots of mean SUZ12 levels in 1 kb windows of SUZ12 peaks quantified by qChIP-seq. Two-sided Wilcoxon signed-rank test *P* values. *n* = 3 biological replicates. **c-g**, Box plots as in Fig. 2a. **h**, Venn diagrams illustrating strong overlap of SUZ12 peaks in WT, MCM2-2A#1 and MCM2-2A#2 clones (*n* = 3 biological replicates). **i**, Individual SUZ12 SCAR-seq replicates related to Fig. 2j, including two MCM2-2A clones. H3K27me3 SCAR-seq was performed in parallel as control for crosslinked SCAR-seq. SCAR-seq is represented as in Fig. 1.

To reveal global changes in histone modifications in MCM2-2A ESCs, we quantified histone H3–H4 methylation and acetylation levels by mass spectrometry (MS; including H3K4me1/H3K4me2/H3K4me3/H3K4ac, H3K9me1/H3K9me2/H3K9me3/H3K9ac, H3K14/H3K18/H3K23ac, H3K27me1/H3K27me2/H3K27me3/H3K27ac, H3K36me1/H3K36me2/H3K36me3/H3K36ac, H3K79me1/H3K79me2/H3K79me3/H3K79ac and H4ac1/H4ac2/H4ac3/H4ac4 and H4K20me1/H4K20me2/H4K20me3). Overall, changes in histone PTM levels were modest, although there was a general trend toward reduced acetylation and gain of methylation (Extended Data Fig. 4a–f). If H3K9me3 and H3K27me3 propagate only through read-write mechanisms in absence of establishment activity, the modifications would be lost across successive cell divisions in MCM2-2A cells. This was not the case with respect to global levels, rather MCM2-2A cells showed an unexpected gain of H3K27me3 (Fig. 2a). We thus explored changes in histone PTM occupancy and included a rescue cell line (MCM2-R) with the MCM2-2A mutations reverted to WT sequence to separate direct effects of defective histone recycling from clonal differences and new stable epigenetic states that might arise in MCM2-2A cells. Notably, symmetric histone recycling was restored in MCM2-R cells (Fig. 2b), and the global gain of H3K27me3 was rescued (Fig. 2c). To accurately dissect genome-wide changes in histone PTM occupancy, we used a window-based quantitative approach as H3K27me3 showed elevated signal outside peaks in MCM2-2A cells that influenced peak calling (Extended Data Fig. 5a). The H3K27me3 signal was redistributed from high/medium-level regions to low-level regions, and this was rescued in MCM2-R cells (Extended Data Fig. 5b). The increase in low-level H3K27me3 was most pronounced in early replicating regions (Fig. 2d and Extended Data Fig. 5c), including H3K36me2/H3K36me3-marked intergenic and gene body regions (Fig. 2e and Extended Data Fig. 5d) where H3K27me3 gain was mirrored by loss of H3K27ac (Extended Data Fig. 5e). This suggests that H3K27me3 spreading in part reflects lack of negative feedback^18^ from other histone PTMs such as H3K36me2/H3K36me3 (refs. ^36–39^) and H3K4me3 (refs. ^18,38,40^) that are also asymmetrically segregated (Fig. 1d; ref. ^14^) and thus depleted on the blank lagging strand in MCM2-2A cells.

In addition to the increased genome-wide low-level signal, MCM2-2A cells showed changes in H3K27me3 distribution across H3K27me3 domains, mainly at promoters, and this was rescued in MCM2-R cells (Fig. 2f,g). Overall, gains were larger in magnitude than losses (Extended Data Fig. 5f), arguing that the global H3K27me3 increase results from both unscheduled genome-wide increase and gains in H3K27me3 domains. Binding of the PRC2 subunit SUZ12 was also increased (Extended Data Fig. 5g), and H3K27me3 gain at promoters correlated with SUZ12 gain (Fig. 2h). Overall, SUZ12 binding sites were maintained in MCM2-2A cells (Extended Data Fig. 5h), but there was a redistribution from high-occupancy sites to lower-occupancy sites (Fig. 2i). This argues that asymmetric segregation of histones impacts on SUZ12 occupancy, and we thus investigated SUZ12 recruitment post-replication by SCAR-seq. SUZ12 was recruited to newly replicated DNA in a highly symmetrical manner in WT cells but showed a modest lagging strand bias in MCM2-2A cells, despite H3K27me3 segregation to the leading strand (Fig. 2j and Extended Data Fig. 5i). Therefore, PRC2 is not recruited by H3K27me3 post-replication, consistent with its interaction with chromatin being largely independent of H3K27me3 binding (refs. ^41,42^) and mediated in part by DNA^43,44^. Nonetheless, parental histone segregation influenced SUZ12 recruitment post-replication in a manner that favored binding on the blank lagging strand. Collectively, this argues that symmetric histone recycling limits H3K27me3 noise across the genome and focuses PRC2 activity toward high-occupancy sites that are generally cell-type specific.

**Fig. 3:**
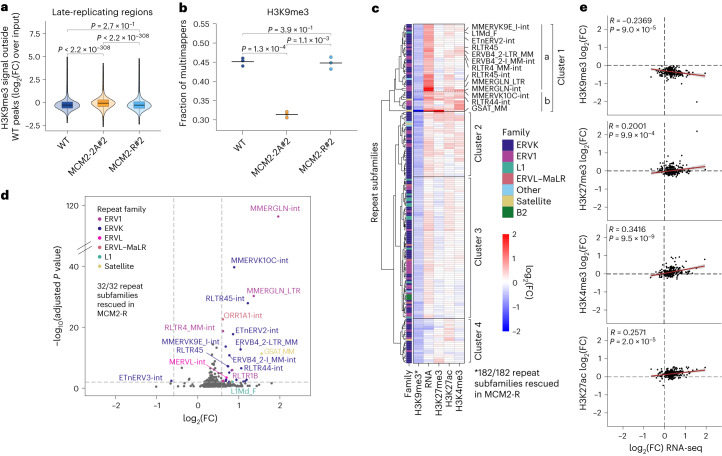
Unscheduled H3K9me3 gains in late-replicating regions and loss of H3K9me3-mediated repeat repression in MCM2-2A cells. **a**, H3K9me3 ChIP–seq signal in 5-kb bins outside WT peaks focused on late-replicating regions. Two-sided Wilcoxon signed-rank test. **b**, Fraction of multimapping reads for H3K9me3 ChIP–seq. *n* = 3 biological replicates. Horizontal lines represent mean values. Two-sided paired *t* test. **c**, Repeat subfamilies with significant H3K9me3 loss in MCM2-2A#2, (*n* = 182 repeat subfamilies) FDR < 0.01, Wald test. Hierarchical clustering according to changes in H3K9me3, RNA, H3K27me3, H3K27ac and H3K4me3 levels between WT and MCM2-2A#2. Selected upregulated repeat subfamilies are labeled. *indicates the number of rescued H3K9me3 repeat subfamilies. **d**, Differential repeat expression between MCM2-2A#2 and WT. Significant subfamilies |log_2_(FC)| > 0.58, FDR < 0.01, Wald test (Supplementary Methods) are colored according to repeat family. *n* = 4 biological replicates. **e**, Correlation of RNA and histone PTM changes (log_2_(FC) MCM2-2A#2/WT) for repeat subfamilies with a significant change in RNA or histone PTMs (*n* = 124; FDR < 0.01). Two-sided Pearson’s correlation coefficient (*R*) with *P* value. Error bands, confidence intervals around the mean. FC, fold change.

**Extended Data Fig. 6:**
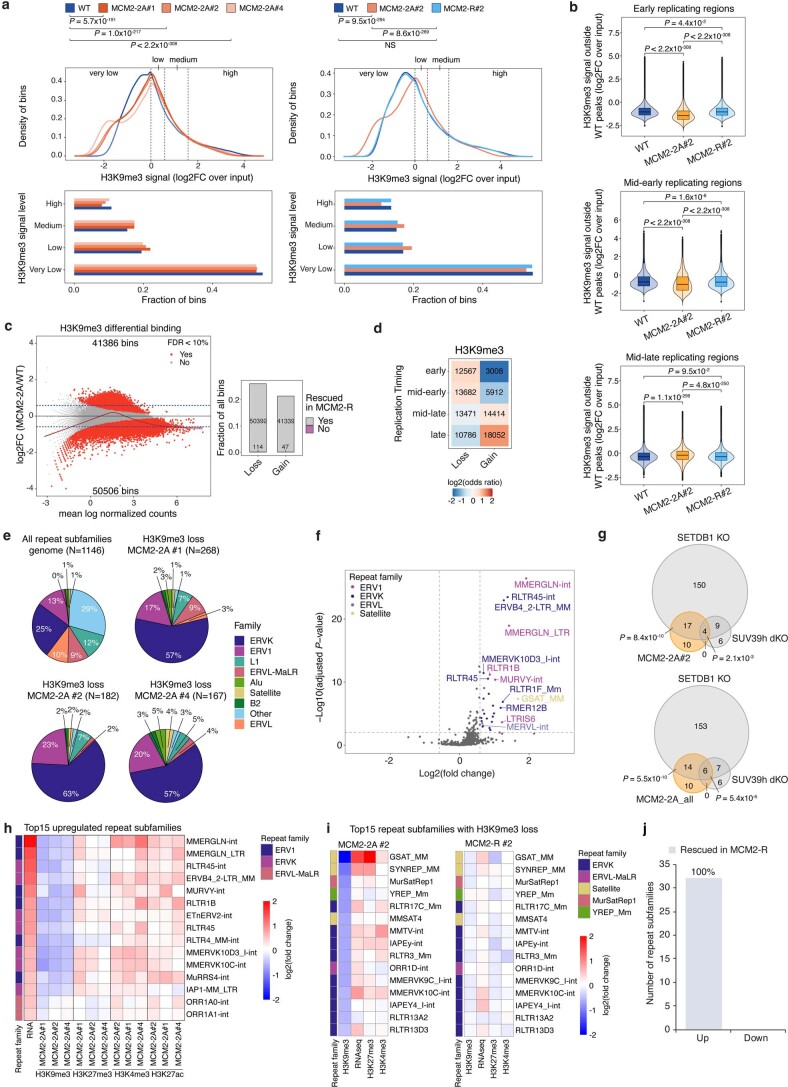
H3K9me3 loss and repeat activation in MCM2-2A. **a**, Density plot showing H3K9me3 signal distribution in genome-wide 5-kb bins (top). Two-sided Wilcoxon signed-rank test *P* values. Quantification of bins across signal categories (bottom). Three MCM2-2A clones (left; average of *n* = 2 biological replicates) demonstrate similar trends across clones and rescue in MCM2-R (right; average of *n* = 3 biological replicates) **b**, H3K9me3 signal in bins non-overlapping WT peaks across replication timing, related to Fig. 3a. Two-sided Wilcoxon signed-rank test *P* values. *n* = 3 biological replicates. Box plots as in Fig. 2a. **c**, H3K9me3 differential occupancy (DO) in MCM2-2A#2 versus WT in 5-kb bins overlapping H3K9me3 WT peaks (left) and bar plot showing rescue in MCM2-R#2 (right). Significant DO (red), FDR < 0.1, Bayes quasi-likelihood F-test (see Supplementary Methods). *n* = 3 biological replicates. **d**, Enrichments analysis (odds ratios) of H3K9me3 DO according to replication timing. Significant states (*P* value < 0.001, two-sided Fisher’s exact test) are colored according to enrichment (red) or depletion (blue), NS states are shown in gray. *n* = number of bins. **e**, Repeat subfamilies with significant loss of H3K9me3 in three MCM2-2A clones (FDR < 0.01, Wald test). **f**, Differential repeat expression between MCM2-2A and WT cells. Significant subfamilies (|Log2FC|> 0.58, adjusted *P* value < 0.01, Wald test) are colored according to repeat family. Fold change (FC) against FDR is shown per repeat subfamily. *n* = biological replicates; WT#1 (*n* = 5); WT#2, WT#3, WT#4, WT#6, WT#7, WT#8, MCM2-2A#4, MCM2-2A#5, MCM2-2A#6, MCM2-2A#7, MCM2-2A#8 (*n* = 2); WT#5, MCM2-2A#2, MCM2-2A#3 (*n* = 3); MCM2-2A#1 (*n* = 6). **g**, Overlap of upregulated repeat subfamilies between MCM2-2A#2 (top) or all MCM2-2A clones (bottom) and SETDB1-KO^48^ and SUV39h1/2-dKO^47^. One-sided hypergeometric test *P* values. **h, i**, Heatmap showing relative H3K9me3, H3K27me3, H3K4me3, H3K27ac levels; **h**, for three MCM2-2A clones at the top 15 significantly upregulated repeat subfamilies from **f. i**, Top 15 repeat subfamilies with significant loss of H3K9me3, FDR < 0.01, Wald test. Related to Fig. 3c. **j**, Bar plot showing differentially expressed repeat subfamilies related to Fig. 3d rescued (gray) in MCM2-R. MCM2-2A had *n* = 32 upregulated repeat subfamilies and all repeat expression was rescued. Source data

H3K9me3 occupancy was also altered in MCM2-2A cells with redistribution from high-level regions to medium/low-level regions (Extended Data Fig. 6a). The low-level H3K9me3 signal increased mainly in the late-replicating genome where H3K9me3 is normally enriched (Fig. 3a and Extended Data Fig. 6b), suggesting an increase in sporadic H3K9me3 deposition restricted by compartments. In addition to spreading outside peaks, MCM2-2A cells also showed a redistribution of signal across H3K9me3 occupancy sites found in WT cells, with a strong trend toward gains in late-replicating regions (Extended Data Fig. 6c,d). These changes were in part driven by a major loss from repetitive regions and a concomitant gain in the nonrepetitive genome, illustrated by a redistribution of multimapping reads to unique reads (Fig. 3b) while the total H3K9me3 levels were maintained (Fig. 2a). H3K9me3 loss across repeats was most prominent for the long terminal repeat (LTR) families ERVK and ERV1 (Fig. 3c and Extended Data Fig. 6e), and several of these repeat subfamilies were upregulated (Fig. 3c,d and Extended Data Fig. 6f), consistent with a previous study^16^. H3K9me3 loss did not always cause derepression (Fig. 3c, cluster 4) likely due to DNA methylation^45,46^ or lack of activating input. Upregulated repeats overlapped substantially with repeat expression in cells lacking SUV39h1/SUV39h2 (ref. ^47^) and SETDB1 (ref. ^48^; Extended Data Fig. 6g), although a broader range of repeats is deregulated upon SETDB1-KO. Moreover, repeat expression correlated with loss of H3K9me3 and gain of active histone modifications (Fig. 3e and Extended Data Fig. 6h). Surprisingly, gain of H3K27me3 also correlated with repeat expression (Fig. 3c,e, cluster 1b and cluster 2), suggesting cells attempt to compensate for H3K9me3 loss as described in other settings^49^. Notably, the loss of fidelity in the H3K9me3 landscape was fully rescued in MCM2-R cells (Fig. 3a and Extended Data Fig. 6a–c), including H3K9me3 repeat silencing (182/182 repeat subfamilies with H3K9me3 loss/rescued; 32/32 repeat subfamilies upregulated/rescued; Fig. 3b–d and Extended Data Fig. 6i,j). Collectively, this indicates that histone recycling is required to maintain H3K9me3 in repressed repetitive regions and reduce unscheduled H3K9me3 in the unique genome. The latter might reflect less engagement of H3K9me3 enzymes in read–write activity at repetitive regions, liberating more enzymes to act spuriously in the B compartment.

### Asymmetric histone recycling challenges bivalent genes

**Fig. 4:**
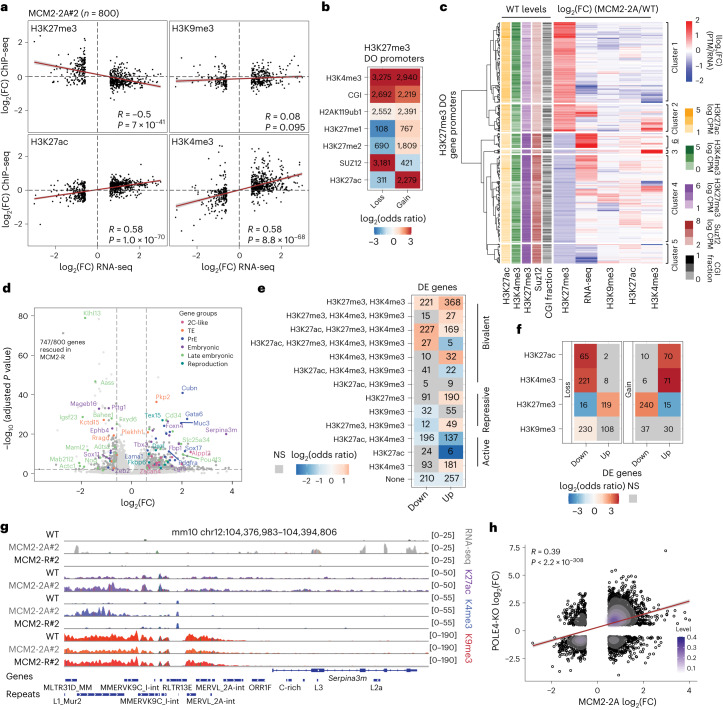
Deregulation of H3K27me3 at bivalent promoters correlates with misexpression of developmental genes in MCM2-2A cells. **a**, Correlation of RNA and histone PTM changes (log_2_(FC) MCM2-2A#2/WT) for DE genes (**d**, *n* = 800). Two-sided Pearson’s correlation coefficient (*R*) with *P* value. **b**, Enrichments (odds ratios) of chromatin feature overlapping H3K27me3 DO promoters. Significant enrichments (*P* < 0.001) are colored, and NS are in gray. Two-sided Fisher’s exact test. **c**, Hierarchical clustering of H3K27me3 DO promoters according to changes in H3K27me3, RNA, H3K9me3, H3K27ac, H3K4me3 between MCM2-2A#2 and WT. WT levels of H3K27ac, H3K4me3, H3K27me3, SUZ12 and CGI are included on the left. *n* = 3,170 H3K27me3 DO promoters. **d**, Differential gene expression between MCM2-2A#2 and WT cells. Significant genes are depicted in dark gray (FDR < 0.01 and |log_2_(FC)| > 0.58, Wald test), and nonrescued genes are depicted in light gray. Selected genes related to enriched GO terms are colored (Extended Data Fig. 7j). FC against FDR is shown per gene. *n* = 4 biological replicates. **e**, Enrichment (odds ratios) of chromatin states around transcription start sites (TSSs) of upregulated and downregulated genes. Significant enrichments are colored (log odds ratio; two-sided Fisher’s exact test, *P* < 0.05), and NS states are in gray. **f**, Histone PTM losses or gains (DO, FDR < 0.01) in DE gene promoters (from **d**). Significant enrichments (*P* ≤ 0.01; two-sided Fisher’s exact test) are colored, and NS states are in gray. **g**, Example region showing upregulation of the *Serpina3m* gene and repeats located within 10 kb, including RNA-seq, H3K27ac, H3K4me3 and H3K9me3 normalized signal. Chr12: 104376983–104394806. **h**, Correlation of gene expression changes (log_2_(FC) over WT) in POLE4-KO and MCM2-2A histone recycling mutants. Single genes and gene densities are represented by black circles and purple color gradient, respectively. Two-sided Pearson’s correlation coefficient (*R*) with *P* value. Source data

**Extended Data Fig. 7:**
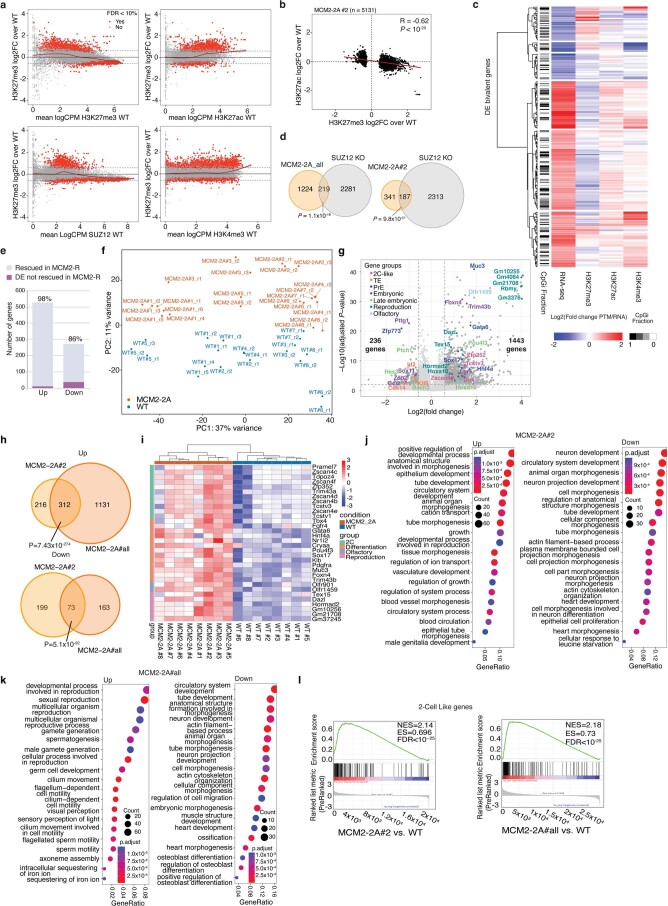
H3K27me3 changes at gene promoters correlate with differential gene expression in MCM2-2A cells. **a**, MA plots showing H3K27me3 DO in MCM2-2A#2 relative to the H3K27me3, SUZ12, H3K27ac and H3K4me3 signal in WT cells. H3K27me3 DO was analyzed in 5-kb bins overlapping H3K27me3 WT promoters and H3K27me3, SUZ12, H3K27ac and H3K4me3 signal is depicted as mean log_2_ counts per million (CPM). *n* = 3 biological replicates. **b**, Correlation of H3K27me3 and H3K27ac DO at promoters with DO of H3K27me3 (*n* = 5131 DO H3K27me3 bins). Two-sided Pearson’s product moment correlation coefficient. **c**, Hierarchical clustering of differentially expressed (DE) genes bivalent promoters (MCM2-2A#2; *n* = 244) according to changes in RNA, H3K27me3, H3K9me3, H3K27ac and H3K4me3 between MCM2-2A#2 and WT. **d**, Overlap of upregulated genes between all MCM2-2A clones (left; MCM2-2A#all, see **g**) or MCM2-2A#2 (right) with SUZ12-KO DE genes^53^. One-sided hypergeometric test *P* values. **e**, Bar plot showing number of DE genes in MCM2-2A rescued (gray) or not rescued (purple) in MCM2-R, related to Fig. 4d. Percentage of rescued genes are indicated. **f**, PCA plot showing a shared expression deviation of 8 MCM2-2A clones (orange) from 8 WT clones (blue) based on all expressed genes in MCM2-2A and WT. *n* = biological replicates; WT#1 (*n* = 5);WT#2, WT#3, WT#4, WT#6, WT#7, WT#8, MCM2-2A#4, MCM2-2A#5, MCM2-2A#6, MCM2-2A#7, MCM2-2A#8 (*n* = 2); WT#5, MCM2-2A#2, MCM2-2A#3 (*n* = 3); MCM2-2A#1 (*n* = 6). **g**, Differential expression analysis of WT and MCM2-2A clones in **f**. Selected gene are in color as indicated. Significant genes (| log2 FC | > 0.58, FDR < 0.01, Wald test) are in dark gray. FC against FDR is shown per gene in MCM2-2A versus WT. **h**, Overlap of DE genes between MCM2-2A#2 (see Fig. 4d) and MCM2-2A#all (see **g**). One-sided hypergeometric test *P* values. **i**, Heatmaps showing relative expression of selected differentially expressed genes in each of the analyzed clones. Values depict log_2_FC from the average of all samples. **j-k**, Dot plot of GO term enrichment analysis in the upregulated and downregulated MCM2-2A#2 genes (**j**) or MCM2-2A#all (**k**) (Related to Fig. 4d and g, respectively). **l**, 2C-like genes^84^ are enriched both in MCM2-2A#2 (left, see Fig. 4d) and in the shared MCM2-2A#all (right, see **g**) upregulated genes in gene set enrichment analysis (GSEA).

To dissect how chromatin alterations in MCM2-2A cells affect gene regulation, we focused on differential histone PTM occupancy at promoters. Loss and gain of H3K27me3 across promoters correlated inversely with changes in gene expression (Fig. 4a), while differential H3K9me3 occupancy did not. The latter is consistent with H3K9me3 loss in repetitive regions, and H3K9me3 gain mainly in the repressed B compartment (Extended Data Fig. 6d). Differential gene expression correlated positively with changes in active modifications (Fig. 4a). However, given that transcription is highly predictive for these modifications, it is difficult to establish cause and effect here^50,51^. We, therefore, focused on all promoters showing differential occupancy (DO) of H3K27me3 (Fig. 2g) to understand the relationship to differential gene expression. These promoters were enriched for bivalent^52^ chromatin states marked by H3K4me3 and H3K27me3 (Fig. 4b). Bivalent promoters showed both loss and gain of H3K27me3, with gains occurring at promoters characterized by H3K27ac and lower H3K27 methylation levels (me1 and me2; Fig. 4b). Consistent with this, unbiased clustering showed H3K27me3 redistribution from promoters with high SUZ12/H3K27me3 to lower-occupancy promoters with more H3K27ac and H3K4me3 (Fig. 4b,c and Extended Data Fig. 7a). The redistribution was not a re-allocation between CpG islands (CGI) and non-CGI promoters (Fig. 4b,c). Collectively, this implies that asymmetric histone segregation challenges the balance of active and repressive modifications at bivalent promoters. Differential H3K27me3 occupancy correlated inversely with transcription for most, but not all, clusters, and H3K27me3 loss was associated with the gain of H3K27ac (Fig. 4c and Extended Data Fig. 7b,c). This argues that changes in H3K27me3 in part underlie differential gene expression in MCM2-2A cells. In agreement, genes upregulated in MCM2-2A and SUZ12-KO cells^53^ overlapped substantially (Extended Data Fig. 7d).

Next, we focused more broadly on differentially expressed (DE) genes in MCM2-2A cells. We identified 800 DE genes, which were largely rescued in MCM2-R cells (93%, 747/800; Fig. 4d and Extended Data Fig. 7e) consistent with re-establishment of H3K9me3 and H3K27me3 landscapes upon restoration of symmetric histone recycling (Figs. 2c–f and 3b–d and Extended Data Figs. 5b–d and 6a–c,i). Moreover, similar gene expression changes were observed across multiple MCM2-2A clones (Extended Data Fig. 7f–i). To identify chromatin states sensitive to asymmetric recycling, we performed a chromatin-state analysis of DE genes in WT cells. DE genes were depleted for active states marked by both H3K4me3 and H3K27ac (Fig. 4e), arguing that histone recycling has a limited impact on active genes. However, upregulated and downregulated genes were strongly enriched for bivalent chromatin states (Fig. 4e), as expected from the DO of H3K27me3 at these promoters (Fig. 4b,c). Accordingly, developmental processes were enriched among DE genes (Extended Data Fig. 7j,k). Upregulated genes were also enriched for repressed chromatin states and included reproduction and 2-cell-like cell (2CLC) gene signatures (Fig. 4d,e and Extended Data Fig. 7j–l). The loss of H3K27me3, not H3K9me3, was predictive for upregulation, while the gain of H3K27me3, not H3K9me3, was predictive for downregulation (Fig. 4f). This supports that DE is linked to H3K27me3 changes in promoters. Furthermore, derepressed repeats were enriched in proximity to about 5% of the upregulated genes (odds ratio = 2.7, *P* = 1 × 10^−12^; two-sided Fisher test), including *Tbx3*, *Serpina3m* and 2CLC genes like *Zscan4* and *Cyp2b23* (Fig. 4g). This suggests that loss of H3K9me3 contributes to gene expression changes in MCM2-2A cells through activation of repeats, as previously described for SETDB1-KO cells^48^ and regulation of pluripotency factors and 2CLC genes^54–56^.

**Extended Data Fig. 8:**
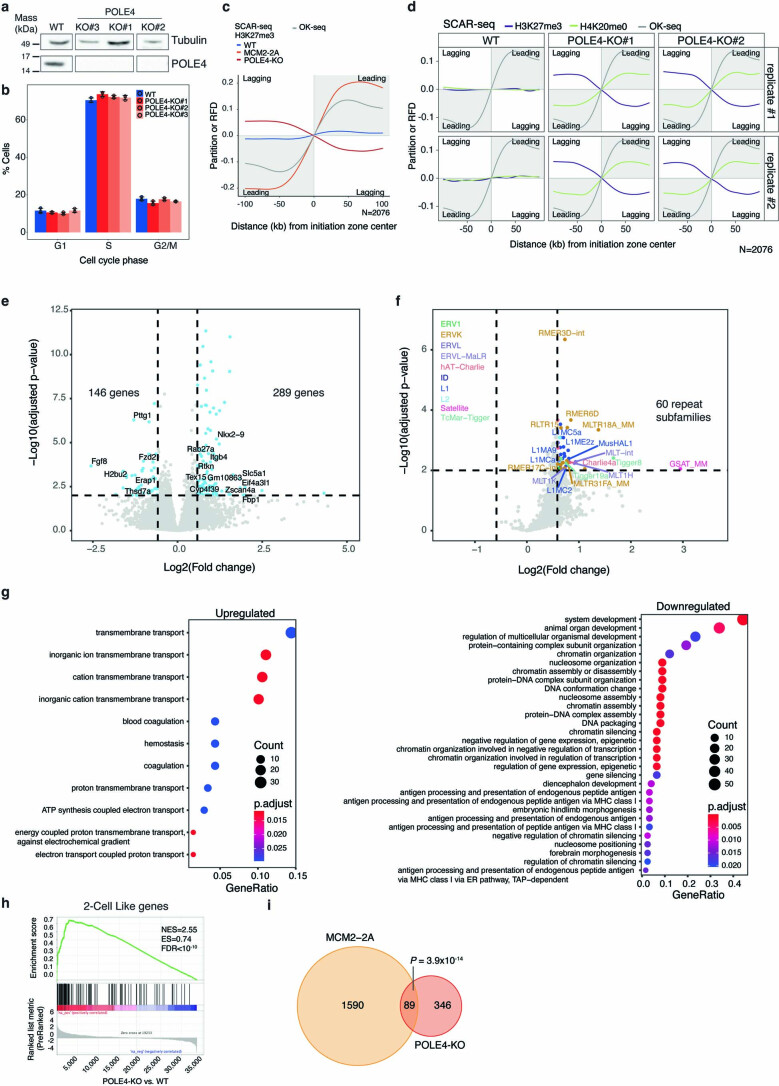
POLE4-KO ESCs shared expression changes with MCM2-2A. **a**, Western blot analysis of POLE4-KO clones. **b**, Cell cycle distribution in three POLE4-KO ESC clones is similar to WT. Bar plot shows the fraction of cells in G1, S and G2/M phase based on flow cytometry analysis of DNA content and EdU labeling. Mean ± s.d. of *n* = 3 biological replicates is shown, dots depict individual data points. **c**, SCAR-seq profiles of H3K27me3 in POLE4-KO, MCM2-2A and WT cells represented as in Fig. 1. **d**, SCAR-seq analysis of nascent chromatin showing lagging strand bias of old histones (H3K27me3) and leading strand bias of new histones (H4K20me0) in two independent POLE4-KO clones. SCAR-seq average profiles are shown as in Fig. 1. **e**, Volcano plot showing differential expression analysis. FC against FDR is shown per gene in POLE4-KO versus WT. Significant genes | log2 FC | > 0.58, FDR < 0.01, Wald test, are depicted in light blue. *n* = biological replicates; WT#1 (*n* = 3), POLE4-KO#1 (*n* = 3), POLE4-KO#2 (*n* = 3), POLE4-KO#3 (*n* = 3). **f**, Volcano plot showing differential repeat expression between POLE4-KO to WT. FC against FDR is shown per repeat subfamily-*n*. Significant subfamilies | Log2FC|> 0.58, FDR < 0.01, Wald test, are colored according to repeat family. *n* as in **e**. **g**, Dot plot of GO term enrichment analysis of biological processes in the downregulated genes and upregulated genes. **h**, Gene set enrichment analysis (GSEA) of 2C-like genes^84^ among POLE4-KO upregulated genes. **i**, Venn diagram illustrating overlaps of DE genes in POLE4-KO and MCM2-2A cells. *P*-values, one-sided hypergeometric test. Source data

To test whether DE in MCM2-2A mutants is caused by asymmetric histone segregation or other potential functions of MCM2 histone binding, including interaction with other chaperones like ASF1 (refs. ^57,58^), we generated POLE4 knockout (KO) ESCs (Extended Data Fig. 8a,b). As expected^16^, these mutants showed asymmetric segregation of parental histones toward the lagging strand, opposite to the leading strand bias of MCM2-2A cells although with lower amplitude (Extended Data Fig. 8c,d). In accordance, POLE4-KO cells showed similar, although less pronounced, gene expression changes as MCM2-2A cells, including upregulation of repeats and 2CLC genes (Fig. 4h and Extended Data Fig. 8e–i). Collectively, this argues that symmetric histone recycling is required to maintain proper H3K27me3 regulation of bivalent genes and H3K9me3-mediated repeat repression.

### Histone recycling underpins cell-state transitions in ESCs

**Fig. 5:**
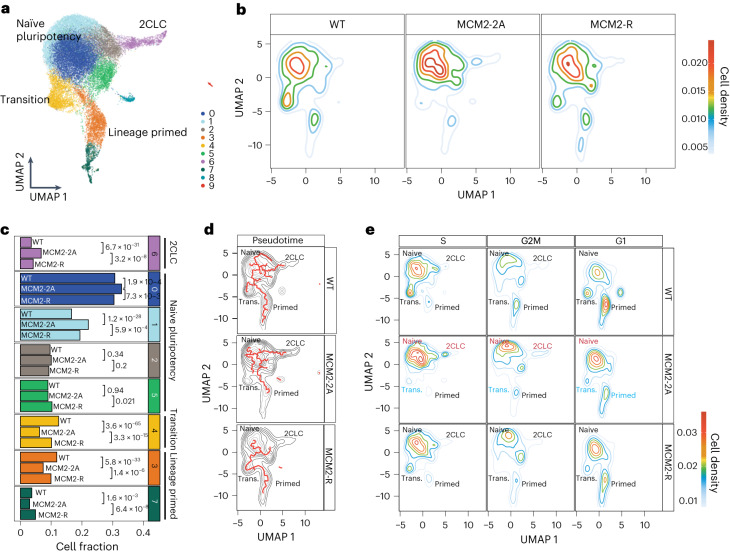
MCM2-2A mutation challenges cell-state transitions. **a**, UMAP showing clustering analysis and annotations of WT, MCM2-2A and MCM2-R cells. **b**, WT, MCM2-2A and MCM2-R cells projected on the common UMAP. Colored lines represent cell density. **c**, Relative abundance of main subpopulations shown in **a** for each cell line. *P* values were derived from chi-square tests comparing cell counts for the clusters of interest with the cell counts of all other clusters. *n* = number of cells; WT (*n* = 15,181), MCM2-2A#2 (*n* = 15,488) examined over two biological replicates, MCM2-R#2 (*n* = 4487). **d**, Pseudotime analysis showing main trajectories between clusters. **e**, Cell-cycle-separated single cells projected on UMAP. Colored lines represent cell density.

**Extended Data Fig. 9:**
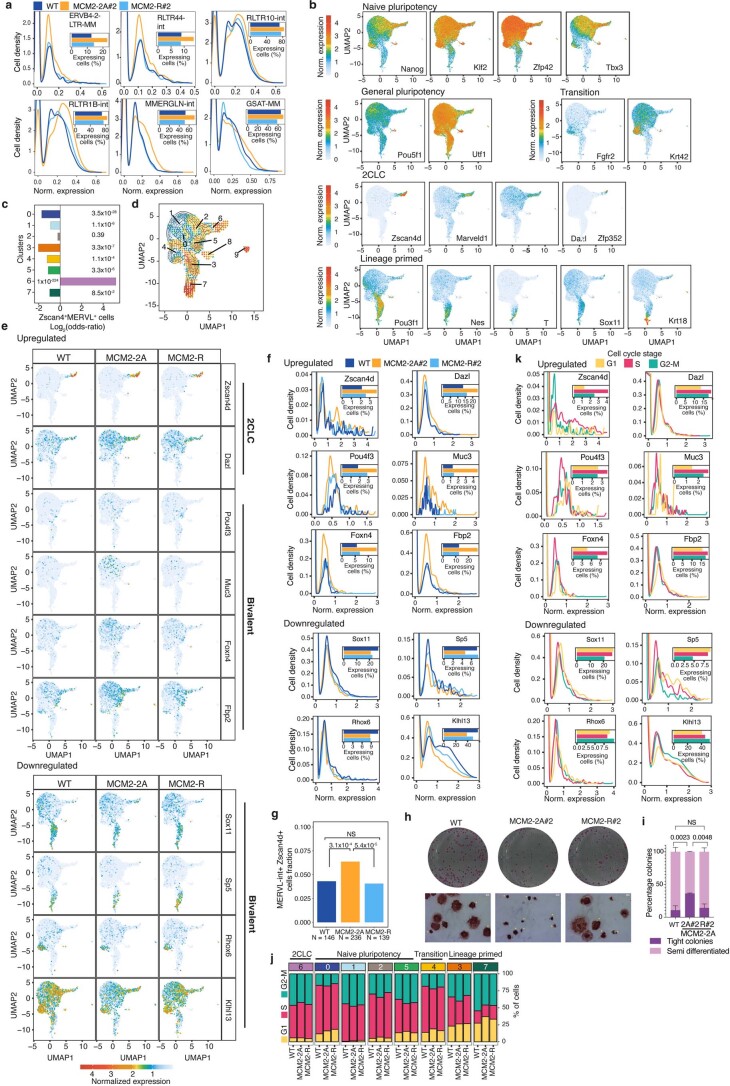
Timely transitions in cellular states are impaired in MCM2-2A cells. **a**, Expression values and cell quantifications for selected repeats DE in the total RNA-seq. Positive WT, MCM2-2A#2 and MCM2-R#2 cells are depicted in bar plot. **b**, UMAP embedding showing expression of gene markers used to classify ESC subpopulation clusters (see Fig. 5a). **c**, Enrichment of *Zscan4* and MERVL double positive cells across the single cell clusters, related to Fig. 5a. **d**, Velocity plot for combined population of all 3 cell lines based on splicing kinetics showing the transcriptional dynamics intrinsic to the trajectories between the different clusters. **e**, WT, MCM2-2A#2 and MCM2-2A-R#2 cells projected on the common UMAP, showing the expression of selected gene markers, bivalent and 2CLC genes DE in total RNA-seq. Bivalent upregulated genes are over-represented in the naïve pluripotency cluster region. **f**, Density plots for genes from **e** across cells in WT, MCM2-2A#2 and MCM2-R#2 clones. Plots as in **a**. **g**, Cells double positive for *Zscan4* and MERVL expression in WT, MCM2-2A and MCM2-R. Chi-square tests *P* values comparing cell counts for each cell line. **h**, Representative images of l 6-wells containing alkaline phosphatase-stained colonies from WT, MCM2-2A#2 and MCM2-R#2 cells. Zoom-in bright-field images (below). Scale bar = 1 mm. Yellow asterisks, tight undifferentiated colonies. **i**, Quantification of colonies from (**h**). Mean percentages are represented with ±s.d., *n* = 3 biological replicates. One-way ANOVA statistical test *P* values. **j**, Cell cycle distribution of single cells in each of the clusters in WT, MCM-2A and MCM2-R displayed as relative cell amount (that is, percentage). Note that the relative cell cycle distribution does not provide information on the total number of cells in each cluster. **k**, Density plots showing expression values and cell quantifications for genes from **e** across all analyzed cells (MCM2-2A, WT and MCM2-R) in the different cell cycle phases G1, S and G2/M. Positive cells in the cell cycle phases G1, S and G2/M are depicted in a bar plot. Source data

To address whether differential gene expression in MCM2-2A cells occurs population-wide or in specific subpopulations, we performed single-cell RNA-seq (scRNA-seq). Upregulation of repeats in single MCM2-2A cells involved both increased expression across the population and a higher fraction of cells expressing certain repeats, with both patterns fully rescued in MCM2-R cells (Extended Data Fig. 9a). Joint uniform manifold approximation and projections (UMAP) and cluster analysis revealed naïve pluripotency clusters, 2CLCs, and lineage-primed clusters in which pluripotency factors such as *Pou5f1* and *Utf1* are co-expressed alongside lineage specifiers like *Pou3f1*, *Nes* and *T* (Fig. 5a and Extended Data Fig. 9b,c), as previously described for serum/Leukemia inhibitory factor (LIF) conditions^4,59,60^. Interestingly, WT and MCM2-R ESCs distributed similarly across this spectrum of cell states (Fig. 5b,c), exploring pluripotency and transiting between canonical naïve states and lineage-primed states (Fig. 5b–d and Extended Data Fig. 9d). In contrast, MCM2-2A cells exhibited less cell-state transitions, mainly populating naïve pluripotency and 2CLC states (Fig. 5b–d). Lineage priming (clusters 3 and 7) and the transition state (cluster 4), linking naïve pluripotency and lineage priming, were substantially reduced in MCM2-2A cells (Fig. 5b–d). These altered population dynamics in MCM2-2A cells are reflected in the upregulation of 2CLC genes and downregulation of differentiation genes linked to lineage priming in the bulk RNA-seq (Fig. 4d and Extended Data Figs. 7g–l and 9e–g) and were also apparent in the lower frequencies with which single cells could spontaneously differentiate in clonal expansion (Extended Data Fig. 9h,i). Together, this argues that chromatin alterations in response to asymmetric histone segregation challenge normal ESC-state transitions, impairing their capacity to exit more naïve states and prime for differentiation.

In MCM2-2A mutants, histone PTM asymmetries are most dramatic in the S phase, and chromatin restoration processes gradually resolve asymmetry as cells progress in the cell cycle (Fig. 1). We, therefore, analyzed changes in transcriptional states across the cell cycle, separating S, G2/M and G1 populations according to a cell cycle assignment algorithm^61^ (Fig. 5e and Extended Data Fig. 9k,j). In S phase, WT cells explored states highly enriched for naïve pluripotent and 2CLC gene expression, and in G2 phase and especially G1 phase, more cells transitioned toward lineage-primed states. This is consistent with previous findings that G1 cells exhibit downregulation of the naïve pluripotency gene regulatory network and higher levels of differentiation genes when compared to S-phase cells^59,62,63^. In contrast, fewer MCM2-2A cells exited naïve pluripotency and explored lineage-primed states in G1. In line with this, the probability of MCM2-2A cells to enter the 2CLC state in S phase was enhanced (Fig. 5e). Moreover, developmental genes sporadically expressed in S-phase cells (not G1) showed increased expression in MCM2-2A (Extended Data Fig. 9k), both in the bulk and single-cell experiments (Fig. 4d and Extended Data Fig. 9e,f). Interestingly, this misexpression happened in the naïve pluripotency cluster (Extended Data Fig. 9e). This suggests that high histone PTM asymmetry between sister chromatids generates more permissive conditions for gene activation, most plausibly on the blank lagging strand. Notably, this propensity was rescued by reverting to symmetric histone segregation in MCM2-R, reducing 2CLC state cells and expression of bivalent genes (Figs. 4d and 5b–d and Extended Data Fig. 9e–g). MCM2-R cells also regained plasticity in terms of increased transitioning into lineage-primed states in the G1 phase (Fig. 5e and Extended Data Fig. 9e–i). This argues that the coordinated dampening of the pluripotency network and expression of lineage priming genes in G1 relies on symmetric histone recycling and proficient post-replication chromatin restoration.

### Histone recycling is required for developmental competence

**Fig. 6:**
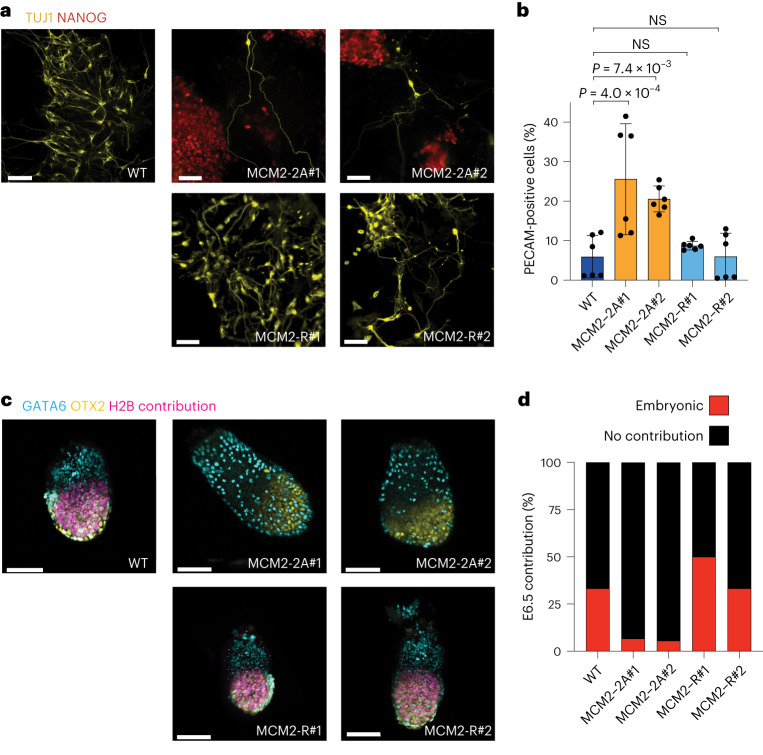
MCM2-2A mutation impairs embryonic differentiation. **a**, Representative immunofluorescence (IF) images of neuronal differentiation (day 7). Scale bar, 70 μm. **b**, Bar plots depicting mean ± standard deviation (s.d.) of PECAM-1-positive cells quantified by flow cytometry at day 7 of neuronal differentiation. One-way ANOVA statistical test. *n* = 6 biological replicates. Dots represent individual data points. **c**, Representative IF images of chimeric embryos from single-cell injected morulae dissected at E6.5. Scale bar, 50 μm. The images are stacks of multiple fields. **d**, Quantification of chimera contribution at E6.5. *n* = number of embryos; WT (*n* = 18), MCM2-2A#1 (*n* = 15), MCM2-2A#2 (*n* = 18), MCM2-R#1 (*n* = 20) and MCM2-R#2 (*n* = 15). ANOVA, analysis of variance. Source data

**Extended Data Fig. 10:**
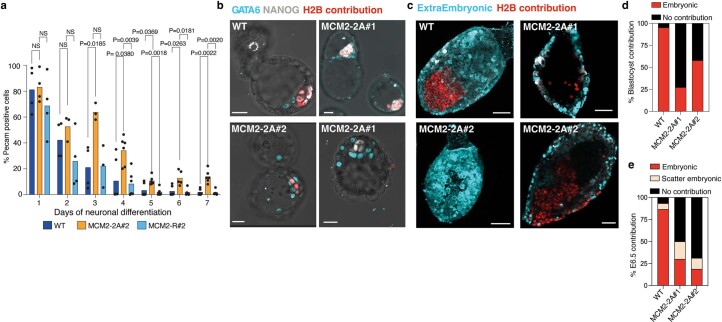
MCM2-2A mutation impairs differentiation in vitro and in vivo. **a**, Bar plots representing the mean of PECAM-1 positive cells quantified by flow cytometry in a 7-day time course of neuronal differentiation. Dots depict individual data points. *n* = 6 biological repeats. Two-way ANOVA Statistical test *P* values. **b**, **c**, Representative IF images of chimeric embryos from 4-cell injected morulae cultured until the hatched blastocyst stage in vitro equivalent of E4.5 (**b**, scale bar, 20 µm) and injected morula at E6.5 (**c**, scale bar 50 µm). (**c**) Images on the left are stacks of multiple fields, images on the right are individual optical sections. Top left and bottom right show embryonic contribution, top right shows scatter embryonic contribution and bottom left image shows an example of no contribution as quantified in **d**. **d**, Quantification of chimera contribution at blastocyst stage from 4-cell injections. *n* = number of embryos; 21, 22, 19, left to right. **e**, Quantification of chimera contribution at E6.5 from 4-cell injections. *n* = number of embryos; 15, 10, 16, left to right. Source data

The reduced plasticity in MCM2-2A ESC cultures suggested that differentiation capacity would be compromised. Indeed, when challenged to undergo neural differentiation, MCM2-2A cells failed to produce morphologically normal TUJ1-positive neurons and aberrantly maintained expression of ESC markers NANOG and PECAM-1 (Fig. 6a,b), consistent with a recent study^58^. MCM2-2A cultures also maintained higher levels of PECAM-1 early after induction of differentiation (Extended Data Fig. 10a). Notably, this phenotype was rescued in MCM2-R cells (Fig. 6a,b and Extended Data Fig. 10a). This, together with reduced lineage priming in ESCs, argues that symmetric histone segregation is necessary for robust transition from pluripotency toward a specific differentiation trajectory. To further probe the impact of histone PTM inheritance on lineage specification, we assessed the capacity of MCM2-2A ESCs to contribute to embryonic development in chimera assays by injecting H2B-mCherry-labeled WT, MCM2-2A and MCM2-R cells into morulae (Fig. 6c,d and Extended Data Fig. 10b–e). Although MCM2-2A mutant cells could incorporate into embryos and were found at the late blastocyst stage (E4.5 equivalent), their capacity to colonize the epiblast was substantially reduced at postimplantation stages (E6.5; Extended Data Fig. 10b–e). As pluri-/totipotency is defined based on the capacity of a single cell to generate all lineages^64,65^, we assessed the capacity of individual MCM2-2A ESCs to colonize E6.5 embryos. Although single WT and MCM2-R cells robustly contributed to postimplantation development, MCM2-2A mutants were unable to do so (Fig. 6c,d). Collectively, these observations argue that symmetric histone segregation is required for the proper exit from pluripotency and efficient embryonic differentiation.

## Discussion

**Fig. 7:**
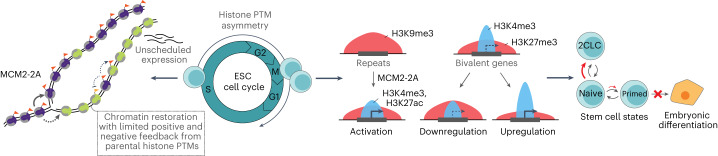
Model illustrating how asymmetric histone segregation challenges epigenome fidelity and ESC functionality. H3–H4 asymmetry in MCM2-2A cells creates a lagging strand largely devoid of parental histone PTMs, which affects the accuracy of chromatin restoration and creates a permissive environment for unscheduled expression in every cell cycle. This broadly alters the histone PTM epigenome with both local and global changes in repressive modifications. MCM2-2A cells show loss of H3K9me3-based repeat repression, misregulation of H3K27me3 and bivalent genes, and reduced ESC plasticity and embryonic differentiation. Naïve, naïve pluripotency; primed, lineage-primed states; new histones, green; parental histones, purple.

Here we show that asymmetric recycling of modified histones H3–H4 in MCM2-2A ESCs results in sister-chromatid imbalances in S/G2 that are, in part, transmitted to daughter cells (Fig. 7). This causes global and local redistribution of H3K27me3 and H3K9me3, arguing that symmetric histone recycling is required to reduce noise and focus the activities of histone-modifying enzymes and hereby underpin correct, balanced chromatin restoration. In MCM2-2A cells, loss of H3K9me3 challenges repression of repeats, while H3K27me3 changes at bivalent promoters correlate with deregulation of developmental genes (Fig. 7). These expression changes are linked to reduced plasticity in MCM2-2A cells, where naïve pluripotent states are favored over lineage priming and developmental competence is reduced (Fig. 7). Restoration of symmetric histone recycling rescues both molecular and developmental phenotypes. Together, this argues that symmetric histone H3–H4 segregation and balanced inheritance of histone PTMs maintain the plastic chromatin environment that underpins ESC identity.

Unspecific deposition of both H3K9me3 and H3K27me3 in MCM2-2A cells suggests that spurious enzyme activity is limited by correct histone recycling. The role of histone recycling in constraining activity of modifying enzymes is in line with negative crosstalk between histone modifications^18^ such as the antagonism between H3K27 and H3K36 methylation^19,36–39^. Symmetric histone recycling is also required to maintain H3K9me3 domains and transcriptional silencing across many repeats, consistent with a *cis* read–write propagation model mirroring Clr4 read–write function in *Schizosaccharomyces pombe* heterochromatin^20,23,24^. Additional repressive layers such as Krüppel-associated box domain zinc finger proteins (KRAB-ZFPs), ncRNA and DNA methylation^5,6,46^ could allow H3K9me3 silencing to be re-established in MCM2-R cells. By challenging recycling—a maintenance component of the multilayered repressive system—MCM2-2A mutation shifts the balance toward activation for LTRs in ESCs as these repeats share an intimate interplay with the pluripotency network^66,67^. Other cell types would likely show different vulnerabilities.

In MCM2-2A mutants, H3K27me3 occupancy and PRC2 binding are reduced at high-occupancy sites while accumulating at lower-occupancy sites. Thus, although H3K27me3 is low on the lagging strand in S/G2, PRC2 almost suffices to establish H3K27me3 domains on the lagging strand in each cell cycle. Consistent with this, PRC2 recruitment to the lagging strand is efficient (this work) and the H3K27me3 landscape can be installed de novo in ESCs^26,68^. We speculate that full maintenance of high H3K27me3 occupancy sites requires efficient read–write activation of PRC2 on both daughter strands and that imbalanced recruitment post-replication favors PRC2 activity at lower H3K27me3 occupancy sites. Allosteric activation of PRC2 is required for the efficient establishment of H3K27me3 in vitro and in ESCs^22,26^. We anticipate that allosteric PRC2 activation could initially be mediated by JARID2 (refs. ^69,70^) on the lagging strand, as JARID2 recruitment is generally high in S/G2 phase^71^. Consistent with this, recycling of H2AK119ub1, recognized by JARID2, can contribute to H3K27me3 establishment on the lagging strand in MCM2-2A cells where H2A–H2B recycling is unaffected^27^. Activation in *trans* from parental H3K27me3 on the leading strand might also occur. However, we conclude that a contribution of parental histone H3–H4 PTMs in *cis*, in every round of chromatin replication, is important for focusing PRC2 activity and fine-tuning H3K27me3 occupancy. In ESCs, bivalent developmental genes are particularly sensitive to misregulation as they are fine-tuned by the balance of H3K27me3 and activating input. Again, other cell types would likely show different vulnerabilities.

Changes in gene expression and ESC subpopulations in MCM2-2A are linked to both chromatin restoration defects (every S phase) and changes in histone PTM landscapes shaped by multiple cell divisions. MCM2-2A cells show increased expression of 2CLC genes and elevated firing of a subset of differentiation genes in the S phase when asymmetry is prevalent. Impaired new histone deposition and lack of repressive histone modifiers also enhance 2CLC gene expression^72,73^, implying that this is a general phenotype of chromatin restoration defects. As in MCM2-2A cells, expression of repeats also promotes 2CLC state and naïve pluripotency in these settings^48,56,74^. This likely explains why MCM2-2A cells show impaired transition toward lineage priming in G1. The defect in exiting pluripotency during neuronal differentiation (this work and ref. ^58^) also aligns with impaired lineage priming and elevated naïve pluripotency in MCM2-2A ESCs. A recent study proposed that MCM2 with the ASF1A histone chaperone directly activates bivalent genes during neuronal differentiation via nucleosome eviction^58^. However, neither MCM2 nor ASF1 has nucleosome disassembly activity in vitro^75,76^, and evidence of recruitment to specific bivalent promoters is limited, while MCM2 function in replication-coupled histone dynamics and histone recycling is well established^13,14,16,28,57,77,78^. Although other roles of MCM2 in histone dynamics could contribute to the MCM2-2A phenotype, gene expression changes in the histone recycling mutants MCM2-2A, POLE4-KO and POLA1-3A are highly correlated (this work and ref. ^16^), including increased expression of 2CLC genes and repeats. This argues that asymmetric histone recycling drives the gene expression changes and differentiation phenotype in MCM2-2A ESCs. Early embryonic development requires highly coordinated changes in gene expression programs with the restructuring of H3K27me3 landscapes and downregulation of repeat expression^3,46,52,67^. The chromatin changes in MCM2-2A ESCs, including redistribution of H3K9me3 and H3K27me3, show this regulation is challenged. Restoration of symmetric histone segregation in MCM2-2A cells rescued the developmental defects, demonstrating that balanced inheritance matters in each and every cell cycle to maintain a fine-tuned epigenome that supports plasticity in ESCs. Our work further suggests that the memory provided by histone recycling is important to reduce epigenetic noise and focus the activity of chromatin-modifying enzymes, which could present a barrier to epigenome decline of relevance to cancer and aging^79,80^.

## Methods

The research in this study was conducted under the ethical approval of the Danish Regulatory Authority under project license 2018-15-0201-01520.

### Cell culture and differentiation assays

WT, MCM2-2A, MCM2-R and POLE4-KO mouse ESCs used in this study were derived from the male, E14JU cell line with a 129/Ola background^81^. Genome editing to generate MCM2-2A, MCM2-R and POLE4-KO cell lines is described in Supplementary Methods.

For genome editing and next-generation sequencing experiments, ESCs were grown on gelatin-coated dishes (0.2%) in serum + LIF conditions at 37 °C with 5% CO_2_. Medium was prepared by supplying DMEM–GlutaMAX–pyruvate (Gibco, 31966-021) with FBS (15%; Sigma-Aldrich, F0392), LIF (homemade), 1× nonessential amino acids (Gibco, 11140-050), 1× penicillin/streptomycin (Gibco, 15140-122) and 2-mercaptoethanol (0.1 µM; Gibco, 31350010). Cells were passaged using Trypsin–Ethylenedinitrilotetraacetic acid (EDTA) (Gibco, 25200-056) or TrypLE (Gibco, 10718463). For differentiation and chimera experiments, E14JUs were cultured on 0.1% gelatin. ESCs were cultured in either serum + LIF or in 2i/LIF (N2B27 (1:1 neurobasal medium; Gibco, 21103-049) and DMEM:F12 (Gibco, 21331-020), B27 (Gibco, 17504-044), N2 (homemade), l-glutamine (2 mM; Thermo Fisher Scientific, 25030024), 2-beta-ME (0.1 µM; Gibco, 31350010)), 3 μM GSK3i (Chir99021: Axon Medchem, 1386), 1 μM MEKi (PD0325901: Sigma-Aldrich, 31966-021)) and LIF. Cells were routinely tested for mycoplasma. For neural differentiation, cells were adapted to 2i/LIF medium. In total, 1 × 10^5^ ESCs were plated in gelatinized six-well plates in N2B27 medium with daily medium changes. Neural differentiation was assessed by flow cytometry analysis of PECAM-1 and imaging of TUJ1 and NANOG (Supplementary Table 2).

### SCAR-seq

#### Native SCAR-seq of histone PTMs

In total, 5 × 10^6^ cells were seeded per 15-cm dish 2 d before EdU labeling and nuclei isolation. In total, 4–5 dishes were seeded per time point to get sufficient material for multiple ChIPs. Cells were pulsed in EdU-containing media (10 μM; Jena Bioscience, CLK-N001-25) for 10 or 15 min, as described in Supplementary Table 1. Nascent samples were collected immediately (T0). Chase samples were washed two times with PBS and incubated in media for 1 h (T1), 3 h (T3) or 8 h (T8) before collecting. Samples were collected in ice-cold PBS by scraping and centrifugation, followed by nuclei isolation. Nuclei were aliquoted, snap-frozen and stored at −80 °C.

For MNase digest, nuclei were counted manually using Kova Glasstic Slides and 2 U MNase (Worthington, LS004797) were added per 1 × 10^6^ nuclei. Digests were performed at 30 °C for 20 min. In total, 35–50 μg of digested chromatin was used per sample and incubated with antibodies in a total volume of 600 μl overnight (see Supplementary Table 2 for antibodies). Magnetic beads (anti-rabbit/mouse IgG Dynabeads; Invitrogen, 11203D/11201D) were added and incubated for 2 h. After three washes each with low-salt wash buffer and high-salt wash buffer, DNA was eluted and purified using the MinElute Reaction Cleanup kit (Qiagen, 28204). Mononucleosomal-sized fragments were isolated by double-sided size selection (0.8–3:1) with AMPure XP beads (Beckman Coulter, A63881). EdU-labeled DNA fragments were biotinylated using Click-iT chemistry. Libraries were prepared using the KAPA Hyper Prep Kit (Roche, KK8504). Biotinylated fragments were captured using Dynabeads MyOne Streptavidin (Invitrogen, 65602), and EdU-labeled strands were isolated by performing NaOH washes. Libraries were amplified in 9–11 PCR cycles. Libraries with mononucleosomal-sized inserts were isolated by double-sided size selection (0.77–0.90:1) with AMPure XP beads, followed by a second cleanup (1:1). Fragment distribution was assessed on a Bioanalyzer using the Hgh Sensitivity DNA kit (Agilent) or a Fragment Analyzer system (Agilent). Stranded input samples were prepared for all cell lines and time points in parallel with SCAR-seq samples. For more detailed information about SCAR-seq, please refer to the step-by-step protocol described in ref. ^82^.

#### Crosslinked SCAR-seq

The SCAR-seq protocol described above was adapted to a crosslinked setup to measure the relative binding of SUZ12 on sister chromatids. Samples for H3K27me3 were prepared in parallel as control. Cells were seeded in 15-cm dishes (4 × 10^6^ cells per dish) 2 d before EdU labeling and nuclei isolation. Four dishes were seeded per time point to get sufficient material for both H3K27me3 and SUZ12 samples. Cells were pulsed in an EdU-containing medium (10 μM; Jena Bioscience, CLK-N001-25) for 30 min and crosslinked with formaldehyde at room temperature for 10 min. Crosslinked cells were snap-frozen and stored at −80 °C. Fixation and nuclei isolation was performed using the truChIP chromatin shearing kit (Covaris, 520155) and a sonicated on a Covaris E220 evolution sonicator according to the manufacturer’s recommendations to obtain fragments of 100–500 bp. In total, 100 μg of chromatin was used for H3K27me3 samples and 200–400 μg of chromatin was used for SUZ12 samples. The initial ChIP reactions were set up in 2-ml tubes with 100 μg of chromatin and incubated with antibody in a total volume of 1.5 ml overnight (see Supplementary Table 2 for antibodies). Magnetic beads (Invitrogen Dynabeads Protein G; 30 μl beads for SUZ12 and 50 μl beads for H3K27me3 per 100 μg chromatin) were blocked with BSA overnight and added to the samples and incubated for at least 2 h. Samples were washed 3× with 1 ml of low-salt radio immunoprecipitation assay (RIPA) buffer and 3×/1× (H3K27me3/SUZ12) with 1 ml of high-salt RIPA buffer (5 min rotation in between washes). DNA was eluted in 100 μl of elution buffer (10 mM Tris–HCl (pH 8.0), 5 mM EDTA (pH 8.0), 150 mM NaCl, 1% SDS) by incubation at 65 °C, 1,200 r.p.m. for 30 min, treated with RNase and Proteinase K, decrosslinked and purified using the MinElute Reaction Cleanup kit (Qiagen, 28204). SUZ12 samples were combined during/after MinElute purification. Libraries were prepared using the KAPA Hyper Prep Kit (Roche). SUZ12, H3K27me3 and stranded input libraries were pooled for Click-iT, respectively, and afterward purified by double-sided size selection (0.56–0.85:1) with AMPure XP beads (Beckman Coulter, A63881). Click-iT reaction, streptavidin capture and strand separation were performed as described for native SCAR-seq above. Libraries were amplified in 8–9 PCR cycles for H3K27me3 and stranded input samples, as well as 11–12 PCR cycles for SUZ12 samples. Postamplification cleanup was performed for native SCAR-seq. For further details, see the step-by-step protocols given in ref. ^82^—collection of material, ChIP and library preparation followed the ChOR-seq protocol (except for the changes listed above), whereas Click-iT reaction, streptavidin capture and strand separation followed the native SCAR-seq protocol.

SCAR-seq samples were sequenced on a NextSeq500 instrument (Illumina). All samples are detailed in Supplementary Table 1. Data processing and analysis of SCAR-seq are detailed in the Supplementary Methods.

### Quantitative ChIP–seq

#### Native ChIP–seq

Native ChIP–seq was performed like native SCAR-seq described above, except for omitting EdU labeling and all associated steps for isolation of labeled DNA strands (that is, Click-iT, streptavidin capture and strand separation). Further adaptations of the protocol are described here. In total, 7–10 μg of mouse chromatin was used per sample and incubated with *Drosophila* chromatin (2–3% spike-in) and antibodies in a total volume of 300 μl overnight (see Supplementary Table 2 for antibodies). Libraries were amplified in four PCR cycles. ChIP inputs were prepared for all samples and replicates in parallel.

#### Crosslinked ChIP–seq

Crosslinked ChIP–seq was performed like crosslinked SCAR-seq described above, except for omitting EdU labeling and all associated steps for isolation of labeled DNA strands (that is, Click-iT, streptavidin capture and strand separation). Further adaptations of the protocol are described here. ChIP reactions were set up in 1.5-ml tubes with 50 μg of mouse chromatin and 1 μg of *Drosophila* chromatin (2% spike-in) and incubated with anti-SUZ12 antibody in a total volume of 1 ml (Supplementary Table 2 for antibodies). Two reactions were set up per cell line for a total of 100 μg starting material, and samples were combined after ChIP washes and decrosslinking during purification with a MinElute Reaction Cleanup kit (Qiagen, 28204) by loading the same column twice. A total of 30 μl of Invitrogen Dynabeads Protein G were added per 50 μg of chromatin. Libraries were amplified in seven PCR cycles. ChIP inputs were prepared for all samples and replicates in parallel.

Samples were sequenced on a NextSeq500 or NextSeq 2000 (Illumina). All samples are detailed in Supplementary Table 1. ChIP–seq data processing and analysis are detailed in the Supplementary Methods.

### MS of histone PTMs

Cells were seeded in 15-cm dishes (5 × 10^6^ cells per dish, one dish per cell line) and collected 2 d later by trypsinization and washing in PBS. Cell pellets (1 × 10^7^ cells) were snap-frozen and shipped on dry ice to EpiQMAx GmbH. Sample preparation and MS analysis were performed according to the EpiQMAx GmbH protocols. Briefly, acid-extracted histones were resuspended in Lämmli buffer and separated by a 14–20% gradient SDS–PAGE, stained with Coomassie (Brilliant blue G-250, 35081.01). Protein bands in the molecular weight range of histones (15–23 kDa) were excised as single band/fraction. Gel slices were destained in 50% acetonitrile/50 mM ammonium bicarbonate. Lysine residues were chemically modified by propionylation for 30 min at room temperature with 2.5% propionic anhydride (Sigma-Aldrich, 8.00608) in ammonium bicarbonate, pH 7.5. Subsequently, proteins were digested with 200 ng of trypsin (Promega, V5111) in 50 mM ammonium bicarbonate overnight and the supernatant was desalted by C18-Stagetips (reversed-phase resin) and carbon Top-Tips (Glygen, TT1CAR) according to the manufacturer’s instructions. After desalting, the eluent was speed vacuumed until dryness and stored at −20 °C until MS analysis.

#### Liquid chromatography-mass spectrometry (LC–MS) analysis of histone modifications

Peptides were resuspended in 17 μl of 0.1% Trifluoroacetic Acid (TFA). A total of 5.0 μl were injected into a nano-HPLC device (Thermo Fisher Scientific, UltimateNano3000) using a gradient from 4% solvent B to 90% solvent B (solvent A 0.1% Formic Acid (FA) in water, solvent B 80% Acetonitrile (ACN), 0.1% FA in water) over 90 min at a flow rate of 300 nl min^−1^ in a C18 Ultra-High Pressure Liquid chromatography (UHPLC) column (Thermo Fisher Scientific, 164534). Data were acquired in parallel-reaction monitoring (PRM)-positive mode using a Q Exactive HF spectrometer (Thermo Fisher Scientific) to identify and quantify specific N-terminal peptides of histone H3 and histone H4 proteins and their PTMs. One survey MS1 scan and nine MS2 acquisitions from the precursor m/z value in the inclusion list was performed. MS1 spectra were acquired in the m/z range 250–1,600 with a resolution of 30,000 at m/z 400 (AGC target of 3 × 10^6^). PRM spectra were acquired with resolution 15,000 to a target value of 2 × 10^5^, maximum injection time (IT) 60 ms, isolation 2 window 0.7 m/z and fragmented at 27% normalized collision energy. Typical mass spectrometric conditions were as follows: spray voltage, 1.5 kV; no sheath and auxiliary gas flow; heated capillary temperature, 250 °C. MS histone PTM analysis and quantification are detailed in the Supplementary Methods.

### RNA-seq

Total RNA was extracted from 5 × 10^6^ cells using RNeasy Plus Mini Kit (Qiagen, 74204) according to the manufacturer’s protocol, and gDNA was eliminated by treatment with the RNase-Free DNase Set (Qiagen, 79254). Quality of RNA was assessed using the 2100 Bioanalyzer RNA 6000 Nano kit (Agilent) or the Fragment Analyzer RNA kit (Agilent), all samples had RNA integrity number (RIN) > 8. Total RNA (500 ng) from each sample was depleted of rRNA using the NEBNext rRNA Depletion kit (NEB, E7405L). Strand-specific RNA libraries were prepared using the NEBNext Ultra Directional RNA Library Prep kit (NEB, E7765s), assessed on the Bioanalyzer High Sensitivity DNA kit (Agilent) or the Fragment Analyzer HS NGS kit to ensure good quality and sequenced paired-end on a NextSeq500 (76 bp) or NextSeq 2000 (100 bp; Illumina) in 2–6 biological replicates. All samples are detailed in Supplementary Table 1. RNA-seq data processing and analysis are detailed in the Supplementary Methods.

### Multiplexed scRNA-seq

For the multiplexed scRNA-seq, 1.5 × 10^5^ cells per six-well were seeded on 0.2% gelatin in serum/LIF medium. Cells were dissociated with TrypLE (Gibco, 10718463), resuspended in PBS + 0.04% BSA and passed through 100-μm cell strainer (pluriSelect, 43-10100-60) to obtain a homogeneous single-cell suspension. Samples were automatically counted (Logos Biosystems, LUNA-FX7) and evaluated for viability and homogeneity, and each sample was divided into two to increase the complexity of the cell multiplexing oligonucleotides in the final library. In total, 1 × 10^6^ cells were labeled with cell multiplexing oligonucleotides following the CellPlex kit (10X Genomics, PN-1000261) protocol. Labeled samples and technical repeats were evenly pooled and recounted reaching ~1,500 cells per μl with cell viability higher than 95% for loading at the chromium controller (10X Genomics). In total, 30,000 cells were loaded on one channel of the chromium Next GEM chip G (10X Genomics, PN-1000127) for the targeted recovery of 20,000 single cells. The multiplexed sample was processed using the Chromium Next GEM Single-Cell 3′ Reagent Kits v3.1 (Dual Index; 10X Genomics, PN-1000269). Both cDNA and final libraries' fragment sizes were determined for quality control using the fragment analyzer (Agilent). Final libraries were sequenced on the Illumina NovaSeq 6000 using the SP flow cell to reach 40,000 reads per cell. All samples are detailed in Supplementary Table 1. scRNA-seq data processing and analysis are detailed in the Supplementary Methods.

### Immunofluorescence

Cells and blastocysts were fixed for 10 min in 4% Paraformaldehyde (PFA;Sigma-Aldrich, 158127) at room temperature and stored in PBST (PBS with 0.3% Triton X-100). Primary antibodies were added at the appropriate concentration (Supplementary Table 2) in PBST with 5% donkey serum (Jackson ImmunoResearch, 017-000-121) and incubated overnight. Incubation was followed by three washes in PBS, and secondary antibodies were then added in PBST. Samples were incubated with the secondary antibody (Alexa Fluor, Molecular Probes) in the dark at room temperature for 3 h. After three washes, samples were stained with DAPI (1:10,000) in PBST. Staining of E6.5 embryos involved longer washes. They were blocked for at least 24 h, and primary antibodies were added overnight. Embryos were then washed overnight, and secondary antibodies were also incubated overnight. Cells and embryos were imaged in three dimensions using a Leica TCS SP8 confocal microscope. Images of cells following differentiation were acquired on a Leica AF6000 widefield microscope. Both Leica microscopes use the LASFX software (version 3.7.3.23245) for image acquisition.

### Flow cytometry

#### Cell cycle analysis

For pulse-chase experiments, cells were seeded in 6-cm dishes (3 × 10^5^ cells per dish) 3 d before EdU labeling and sample fixation. Cells were pulsed in EdU-containing media (10 μM; Jena Bioscience, CLK-N001-25) for 15 min. Dishes were pulsed in a staggered manner in groups of nine to ensure accurate labeling and chase times. Nascent samples were collected immediately (T0). Chase samples were washed once with PBS and incubated in a medium with thymidine (5 μM; Sigma-Aldrich, T1895) for 1, 2, 3, 4, 5, 6, 7 and 8 h (T1–T8). For nascent experiments (T0), cells were seeded in six-well plates (2.5 × 10^5^ cells per well) 2 d before EdU labeling and pulsed for 15 min. For collection, cells were trypsinized, washed in cold PBS, fixed in ethanol (100% of cold ethanol was added drop-wise to a final concentration of 70%, while vortexing samples at low speed) and stored at 4 °C for at least 1 h. Cells were permeabilized with 0.25% Triton X-100 in PBS for 10 min at room temperature. Then 5 × 10^5^ cells were resuspended in 200 μl Click-iT reaction mix with Alexa Fluor 647 azide (Invitrogen, C10340) and incubated for 30 min at room temperature, followed by DNA staining with propidium iodide (10 μg ml^−1^) or DAPI (0.25 μg ml^−1^) and simultaneous RNase A treatment (20 μg ml^−1^) for 30 min at room temperature. All washes were carried out with 1% BSA in PBS. Cells were analyzed on a BD FACS Calibur or LSR Fortessa flow cytometer (at least 10,000 cells were recorded per sample). Data were processed in FlowJo (version 10.7.1) using the gating strategy illustrated in Supplementary Fig. 1.

#### Differentiation

Flow cytometry analysis used conjugated antibodies at the concentrations indicated in Supplementary Table 2. Staining of live cells was done for 20 min at 4 °C in the dark in PBS containing 10% FCS. After washing, the final cell pellet was resuspended in PBS/FCS with DAPI (1:10,000) to exclude dead cells. Cells were analyzed using LSR Fortessa flow cytometer (BD Biosciences), using the FACSDiva (BD Biosciences, version 8) software. Plots were generated using FCS Express 6.0 (DeNovo Software, version 6.0), using the gating strategy illustrated in Supplementary Fig. 2.

### Chimera assays

For chimera assays WT 1, MCM2-2A 1, MCM2-2A 2, MCM2-R 1 and MCM2-R 2 cells were labeled with a randomly integrated constitutive CAG-driven H2B-mCherry fusion^83^. Around 20 clones were picked for each cell line, and one clone was selected based on its signal homogeneity and strength, which was analyzed using the LSR Fortessa flow cytometer (BD Biosciences). Injections were carried out by the Core Facility for Transgenic Mice. Mice were maintained in a 12-h light/12-h dark cycle in the designated facilities at the University of Copenhagen, Denmark, with 52% humidity at 22 °C, and air in the room was changed 8–10 times per hour, according to Danish regulations for animal experiments. Eight C57BL/6NRj female mice (4 weeks) underwent superovulation to obtain morulae by intraperitoneal injection (IP) of 5 IU Pregnant Mare Serum Gonadotropin (PMSG;Sigma-Aldrich) per female and IP injection of 5 IU Human chorionic gonadotropin (hCG; Chorulon, Intervet) 47 h later, followed by overnight mating with C57Bl/6NRj stud males. The following morning, females were monitored for copulation plug formation. Embryos were considered E0.5 on the day of plug detection. Live morulae (E2.5) were cultured in EmbryoMax KSOM (Sigma-Aldrich, MR-121) and 1 or 4 H2B-mCherry WT or MCM2-2A ESCs were injected to the morulae and resultant embryos were cultured ex vivo in KOSM microdrops covered with mineral oil (Nidacon, NidOil). Embryos were either cultured for 3 d in vitro to the equivalent of E4.5 in vivo or transferred to RjOrl:SWISS pseudopregnant CD1 females (*n* = 20, CD1 females for single-cell injections and *n* = 12 four-cell injection, 8–13 weeks old) for further development. Embryos were collected at E6.5. Animal work was carried out in accordance with European legislation. All work was authorized by and carried out under Project License 2018-15-0201-01520 issued by the Danish Regulatory Authority.

### Statistics and reproducibility

ChIP–seq and SCAR-seq experiments were conducted with at least two biological replicates, RNA-seq with at least three biological replicates or two if multiple clones with similar conditions were tested, in accordance with ENCODE guidelines. No statistical method was used to predetermine the sample size of experiments. No data were excluded from the analysis. The experiments were not randomized, and investigators were not blinded to allocation during experiments and outcome assessment. The statistical tests and number of independent experiments (*n*) are indicated in the figure legends. *P* values were corrected for multiple comparisons (FDR) using the Benjamini–Hochberg method. Bar plots and dot plots represent the mean ± s.d. Box plots display median as a line, with boxes representing the first and third quartiles. Whiskers extend 1.5× interquartile range.

### Reporting summary

Further information on research design is available in the Nature Portfolio Reporting Summary linked to this article.

## Online content

Any methods, additional references, Nature Portfolio reporting summaries, source data, extended data, supplementary information, acknowledgements, peer review information; details of author contributions and competing interests; and statements of data and code availability are available at 10.1038/s41588-023-01476-x.

## Supplementary information

Supplementary Information Supplementary Methods, Supplementary Figs. 1 and 2. Supplementary Tables 1–3 and Supplementary References.

Reporting Summary

Supplementary Table 1 Sequencing data generated in this study.

## Data Availability

Sequence data produced in this study have been deposited in NCBI GEO with the accession code GSE154391. Proteomic data produced in this study have been deposited in ProteomeXchange with the accession codes PXD020326 and PXD030364. We reanalyzed the following mouse ESC publicly available sequencing datasets: H2AK119ub1 (GSE132752: GSM3891343 and GSM3891344, inputs GSM3891350, GSM3891351); H3K27me1 and H3K27me2 (GSE127117: GSM3625691 and GSM3625689, input GSM3625706); H3K36me2 (GSE126864: SRR8601997, SRR86019978, SRR86019979, inputs SRR8602003, SRR8602004, SRR8602005); H3K36me3 (ENCODE: GSM6373350 and GSM6373351, inputs GSM4051038, GSM4051039); SUZ12-KO RNA-seq (GSE127804); SETDB1-KO RNA-seq (BioProject PRJNA544540) and SUV39H1/2-dKO RNA-seq (GSE57092). For genome annotations, we used GENCODE vM23 (https://ftp.ebi.ac.uk/pub/databases/gencode/Gencode_mouse/release_M23/gencode.vM23.annotation.gtf.gz). For repeat analysis, we used the subfamily annotations (https://labshare.cshl.edu/shares/mhammelllab/www-data/TEtranscripts/TE_GTF/GRCm38_GENCODE_rmsk_TE.gtf.gz). For SCAR-seq analysis, we used mESC Okazaki fragment sequencing initiation zones^14^ (GSM3290342). For GSEA analysis, we used 2C-like gene list from ref. ^84^. Source data are provided with this paper.

## Source data

Source Data Fig. 2 Statistical source.
Source Data Fig. 4 Statistical source.
Source Data Fig. 6 Statistical source.
Source Data Extended Data Fig. 1 Statistical source.
Source Data Extended Data Fig. 4 Statistical source.
Source Data Extended Data Fig. 6 Statistical source.
Source Data Extended Data Fig. 8 Unprocessed western blots.
Source Data Extended Data Fig. 8 Statistical source.
Source Data Extended Data Fig. 9 Statistical source.
Source Data Extended Data Fig. 10 Statistical source.

## Code availability

Code for sequencing data analyses is available at https://github.com/anderssonlab/Wenger_et_al_2023, 10.5281/zenodo.8152293.
