# Supplementary material for: Symmetric inheritance of parental histones governs epigenome maintenance and embryonic stem cell identity
Source: Nat Genet. 2023 Sep 4;55(9):1567–78. doi: 10.1038/s41588-023-01476-x (PMC10484787; doi:10.1038/s41588-023-01476-x)
Supplement: Supplementary file 1 — Supplementary Methods, Supplementary Figs. 1 and 2. Supplementary Tables 1–3 and Supplementary References. [file 41588_2023_1476_MOESM1_ESM.pdf]

nature genetics

Article

https://doi.org/10.1038/s41588-023-01476-x

Symmetric inheritance of parental histones governs epigenome maintenance and embryonic stem cell identity

In the format provided by the authors and unedited

Supplementary Methods

Genome editing

MCM2-2A mESCs were generated by changing tyrosine 81 and 90 to alanine residues using transcription activator-like effector nucleases (TALENs) and a recombination reporter plasmid[1]. TALENs were assembled using the Golden Gate TALEN cloning kit[2] (addgene #1000000024) and acceptor vectors SV40-ELD and SV40-KKR[1]. Cells were transfected with 400 ng TALEN-EED, 400 ng TALEN-KKR, 100 ng recombination reporter (pRR-Puro or pRR-EGFP) and 1.1 µg single-stranded oligonucleotide donor #1 (IDT) (Supplementary Table 3) using Lipofectamine 3000 reagent (Invitrogen, L3000015). For the experiment with the Puromycin reporter, cells were selected 24 hours post-transfection with Puromycin (2 µg/mL) for 36 hours and seeded sparsely on a 10 cm dish. After one week of culture, individual clones were picked manually with a pipette and each clone was distributed between two 96-well plates (one plate for genotyping, one plate for expansion). For the experiment with the EGFP reporter, GFP-positive cells were sorted into 96-well plates (BD FACSAria III cell sorter) 24 hours post-transfection and cultured for one week before expansion and genotyping. For PCR genotyping, cells were washed with PBS and lysed by adding 20 µL of ESC DNA lysis buffer (10 mM Tris-HCl pH 8.0, 0.5 mM EDTA, 0.5 % Triton-X100) and 0.5 µL of Proteinase K (20 mg/mL; Sigma, P6556) directly into the wells, followed by incubation at 55 °C for 1.5 hours. Proteinase K was inactivated by incubation at 95 °C for 10 min and lysates were directly used for PCR. The genomic region surrounding the sites to be mutated was amplified in a 12 µL PCR reaction using OneTaq Hot Start 2x Master Mix (NEB, M0484L) and primers #1 and #2 (TAG Copenhagen) (Supplementary Table 3). Half of the PCR product was digested with restriction enzyme AccI (NEB, R0161L) in a 20 µL reaction at 37 °C for 1 hour and was analysed by agarose gel electrophoresis

together with the undigested product. Positive clones were verified by sequencing with primer #3 (TAG Copenhagen) (Supplementary Table 3). Clones which did not carry the MCM2 mutations were kept as control clones. Note that WT#1 refers to the parental cell line, while WT#2-8 refers to negative clones from the genome editing.

MCM2-R mESCs were generated by reversing the mutations back to wild-type sequence in two independent MCM2-2A clones, MCM2-2A#1 and MCM2-2A#2. Genome editing was performed as described above, except that CRISPR-Cas9 was used instead of TALENs. Cells were transfected with 900 ng of SpCas9(BB)-2A-Puro (PX459) V2.0 plasmid (addgene #62988) containing sgRNA #1, 100 ng of recombination reporter (pRR-Puro) and 1 μg of single-stranded oligonucleotide donor #2. PCR genotyping was performed as described above. Oligonucleotide sequences are listed in Supplementary Table 3.

POLE4 KO mESCs were generated by CRISPR-Cas9 using the SpCas9(BB)-2A-Puro (PX459) V2.0 plasmid (addgene #62988) with sgRNA #2 and sgRNA #3 (Supplementary Table 3), which target the Pole4 gene at the beginning of exon 1 and the end of exon 2, respectively. Cells were transfected using Lipofectamine 3000 reagent (Invitrogen, L3000015) using 0.5 μg of each sgRNA-plasmid. Cells were sparsely seeded on a 10 cm dish 24 hours post-transfection and selected with Puromycin (2 μg/mL) for 48 h. Thereafter, cells were expanded and genotyped with primers #4, #5 and #6 (as described above) (Supplementary Table 3). Positive clones were analysed by Sanger sequencing with primers #4 and #5 (IDT) and Pole4 knockout was confirmed by Western Blot. For preparation of whole cell extracts, cells were washed with PBS and lysed on plate by adding Laemmli buffer (50m Tris-HCl pH 6.8, 100 nM DTT, 2% SDS, 10% Glycerol, Bromphenol blue), transferred to 1.5 mL tubes and subsequently incubated with Benzonase (25 U, Sigma, 70746-3) at 37 °C

for 1h, followed by heat denaturation. Samples were separated on a 4-12% NuPAGE Bis-Tris protein gel (Invitrogen, NP0321BOX) and transferred to a Nitrocellulose membrane (Amersham, 15239794). Membrane was blocked for 1 h in 5% skim milk (Sigma, 70166) with PBST and incubated with primary antibodies overnight (Supplementary Table 3). Following 3 PBST washes, membrane was incubated with peroxidase-conjugated secondary antibody for 1 h. Blots were incubated for 5 min with SuperSignal™ West Pico PLUS chemiluminescent solution (Thermo Scientific, 34580) and visualized on (ImageQuant LAS 4000, GE Healthcare).

Pulse-SILAC mass spectrometry

Cells were adapted to light SILAC media. For SILAC media, the DMEM and FBS described above for serum+LIF media were replaced with DMEM for SILAC (Thermo Fisher, 88364) and dialyzed FBS (Thermo Fisher, 6400-044), and media was supplemented with light lysine (K0; Sigma, L8662) at 798 μM and light (R0; Sigma, A8094), medium (R6) or heavy (R10) arginine at 398 μM (Cambridge Isotope Laboratories, CLM-2265-H-PK or CNLM-539-H-PK). Cells were seeded in light SILAC media in 15 cm dishes ($4x10^6$ cells per dish, 1 dish per time point and cell line). On the following day, cells were pulsed with medium SILAC media for 3 hours to label new histones, followed by a chase with heavy SILAC media to track old (light) and new (medium) histone dynamics throughout one cell cycle (see scheme in Extended Data Fig. 1a). Samples were harvested immediately after the pulse (0 h) and at several chase time points (4 h, 8 h, 12 h and 16 h). Dishes were washed once with PBS during media changes. For sample collection, cells were trypsinized, washed twice in cold PBS, snap-frozen and stored at -80 °C until further use. Cell pellets were acid extracted according to standard protocols. Extracted chromatin pellets were air-dried, stored at -20 °C until all replicates were collected, and shipped on dry ice to EpiQMAx GmbH. Further sample

preparation and MS analysis were performed according to the EpiQMAx GmbH protocols. Briefly, acid extracted histones were resuspended in Lämmli buffer and separated by a 14-20% gradient SDS-PAGE, stained with Coomassie (Brilliant blue G-250, 35081.01). Protein bands in the molecular weight range of histones (15-23 kDa) were excised as single band/fraction. Gel slices were destained in 50% acetonitrile/50mM ammonium bicarbonate. Lysine residues were chemically modified by propionylation for 30 min at RT with 2.5% propionic anhydride (Sigma, 8.00608) in ammonium bicarbonate, pH 7.5. Subsequently, proteins were digested with 200ng of trypsin (Promega, V5111) in 50mM ammonium bicarbonate overnight and the supernatant was desalted by C18-Stagetips (reversed-phase resin) and carbon Top-Tips (Glygen, TT1CAR) according to the manufacturer's instructions. After desalting, the eluent was speed vacuumed until dryness and stored at -20°C until MS analysis.

LC-MS analysis: Peptides were re-suspended in 17 µl of 0.1% TFA. A total of 5.0 µl were injected into a nano-HPLC device (Thermo Fisher Scientific, UltimateNano3000) using a gradient from 4% B to 90% B (solvent A 0.1% FA in water, solvent B 80% ACN, 0.1% FA in water) over 90 min at a flow rate of 300 nl/min in a C18 UHPCL column (Thermo Fisher Scientific, 164534). Data was acquired in PRM positive mode using a Q Exactive HF spectrometer (Thermo Fisher Scientific) to identify and quantify specific N-terminal peptides of histone H3 and histone H4 proteins and their PTMs. One survey MS1 scan and 9 MS2 acquisitions precursor m/z value in the inclusion list was performed. MS1 spectra were acquired in the m/z range 250-1600 with resolution 30,000 at m/z 400 (AGC target of $3x10^6$). PRM spectra were acquired with resolution 15,000 to a target value of $2x10^5$, maximum IT 60ms, isolation 2 window 0.7 m/z and fragmented at 27% normalized collision energy.

Typical mass spectrometric conditions were: spray voltage, 1.5kV; no sheath and auxiliary gas flow; heated capillary temperature, 250°C.

Mass spectrometry data analysis

Pulse-SILAC: Raw files were searched with the Skyline software[3] against histone H3, H4 and H2A almost unmodifiable peptides KLPFQR, DNIQGITKPAIR and IIPR, respectively. The MS1 signals of these peptides were extracted with a precursor mass tolerance of 5 ppm. The chromatogram boundaries of +2 and +3 charged peaks were validated and the "Total Area MS1" under the first 4 isotopomers was used for relative quantification and comparison between the label groups light, medium and heavy at each time point.

Histone PTMs: Raw files were searched with the Skyline software[3] against histone H3 and H4 peptides and their respective PTMs with a precursor mass tolerance of 5 ppm. The chromatogram boundaries of +2 and +3 charged peaks were validated and the Total Area MS1 under the first 4 isotopomers was extracted and used for relative quantification and comparison between experimental groups. The Total Area MS1 of co-eluting isobaric peptides (i.e., H3K36me3 and H3K27me2K36me1) was resolved using their unique MS2 fragment ions. The averaged ratio of analogous ions (i.e., y7 vs y7) were used to calculate the respective contribution of the precursors [4]to the isobaric MS1 peak.

Relative abundances (percentages) were calculated as in the following example for H3K18 acetylation:

%H3K18ac = (H3K18ac_K23un + H3K18ac_K23ac) / (H3K18un_K23un + H3K18ac_K23unmod + H3K18un_K23ac + H3K18ac_K23ac)

where "ac" indicates acetylation and "un" indicates unmodified.

Genomic data analysis

SCAR-seq

Data processing: Reads were processed and mapped (mm10) with the ENCODE ChIP-seq pipeline as described below. The resulting processed bam files were split into forward and reverse strands according to the SAM flag, using samtools view (version 1.5) -F 20 and -f 16, respectively.

Histone partition: Signal was computed using the analysis scripts related to[5]. For each strand the SCAR normalized signal (CPM) was computed in 1kb bins and smoothed in a uniform blur considering the neighbouring 30 bins on each side. For each 1kb window, the signal from its corresponding SCAR input was subtracted and negative values were set to zero. Input corrected windows with CPM < 0.3 on both strands were filtered out and not considered for further analyses. The final partition score for each 1kb window was calculated as:

Partition = (F - R)/(F + R)

where F and R correspond to the number of normalized and input-corrected reads for the forward and reverse strand, respectively. The partition value relates to the ratio of histones with a specific modification being segregated to the nascent forward (Partition > 0) or nascent reverse (Partition < 0) strand within each window respectively. Extreme partition ratios (values > 0.9999 quantile or < 00001 quantile) were set to the quantile value. Partition signal from each replicate was analysed separately for statistical robustness analyses within each mark and timepoint, while the average partition signal from both replicates was used for visualization purposes.

Okazaki-seq: Replication fork directionality (RFD) scores and filtered initiation zones (IZs) for mESC were taken from[5] and used as focus points to define replication via leading or lagging strand mechanism. The RFD score in Okazaki-seq is calculated like SCAR-seq partition scores but subtracting the forward (F) strand signal from the reverse (R) strand signal instead: RFD = (R - F)/(F + R)

For each mark (H3K4me3, H3K27me3 and H3K27ac) as well as SUZ12, only IZs zones within 100kb of WT ChIP-seq defined peaks were used for further analysis. Initiation zone edges, where the RFD reach local extrema, were determined within 100 kb upstream and downstream of the initiation zone, by selecting the location with minimum and maximum RFD value, respectively. A window size of 200 kb around each initiation zone was chosen based on the average initiation zone size (from upstream to downstream initiation zone 10 edge) (mean size = 112 kb) and the distances to neighbouring initiation zones (mean distance = 359 kb). This left a total of n = 2076, n = 2040 and n = 2063 IZs used for downstream analyses in H3K27me3, H3K4me3, H3K27ac marks respectively, and n = 1241 IZs for SUZ12.

The difference in SCAR-seq partition ratios at initiation zone edges between leading and lagging enriched strands were tested with a Wilcoxon signed-rank test. The test was performed between each pair of SCAR-seq samples, taking all 1kb windows with sufficient coverage, between 10 and 90kb from its nearest initiation zone but not further away than 10kb from the two RFD extrema at both sides from the initiation zone. To compare partition values on both sides of the initiation zones, an adjusted partition score was computed by negating the partition values of all windows upstream of the initiation zone. For H3K27ac analyses, unstranded signal over 200bp bins was computed using the windowCounts function within the csaw R package[6] after filtering windows with FC over input above 1.5 in at least one of the four clones.

Replication timing profiles: Mouse ESC RT[7] data were downloaded from https://www.replicationdomain.com/database.php# (accession Int14787930) and lifted from mouse mm8 to mouse build mm10 using liftOver. Replication domains were classified as early (log2FC > 0) or late (log2FC < 0) and further split into early (late) and mid-early (mid-late) based on their median log2FC values. IZs were then annotated with replication timing by their nearest replication domain classification.

Quantitative ChIP-seq

Data processing: For peak focused analysis reads were processed according to the ENCODE ChIP-seq pipeline (version 1.3.6): adapters and low-quality reads were filtered with cutadapt (version 2.5), reads were mapped to a hybrid mouse (mm10) and fly (dm3) genome with bwa, duplicate reads were removed with picard (version 2.20.7), ENCODE mm10 blacklist regions were masked and only reads with mapping quality above 30 were considered for further downstream analyses. To obtain reference-adjusted reads per-million (RRPMs), spike-in normalization factors for each sample were calculated as in[8]: 1 / Nd where Nd is the number of exogenous (dm3) reads per million. To calculate the total histone levels in each sample, the total number of unique reads (uniquely and multi mapping) were multiplied by their corresponding spike-in normalization factors.

Read coverage for all ChIP-seq samples were computed from filtered bam files using deepTools multiBamSummary (version 3.0) in bins of 1kb and data was fed to deepTools plotCorrelation for creating pairwise Pearson correlations heatmaps, with all biological replicates showing high correlation. Visualization tracks for ChIP-seq data were created using deeptools (version 3.0) bamCompare, averaging both replicates after multiplying them by their corresponding spike-in scaling factors. For analysis of chromatin marks over repeat subfamilies, filtered reads were mapped (mm10) using STAR (version 2.7.1a) with the same

parameters as for RNA-seq but with the addition of –alignIntronMax 1 and alignEndsType EndToEnd. Histone level quantification over repeat subfamilies was obtained with the same program, parameters, and annotations as for RNA-seq (see below).

Peak calling was performed with macs2 (version 2.2.4[9]), narrow peaks for H3K4me3, H3K27ac and SUZ12with parameters –nomodel -p 0.05, while broad peaks (H3K27me3 and H3K9me3 and all external native ChIP datasets were called with danpos (version 2.2.4)[10] dregion with default parameters. All other broad PTMs from external crosslinked datasets were called with macs2 using parameters --nomodel --broad -p 0.01 —broad-cutoff 0.1, using as controls their corresponding pooled inputs from all replicates. For each clone a confident set of peaks was obtained by calling peaks on the pooled replicates and keeping peaks in the pooled set that overlapped by at least 50% bp in at least 2 replicates. To obtain a list of confident nucleation sites, a SUZ12 strict set of peaks for each clone was obtained by irreproducible discovery rate (IDR)[11] analyses with cutoff of IDR < 0.05.
External datasets:
- H2AK119ub1 (GSE132752: GSM3891343 and GSM3891344, inputs GSM3891350, GSM3891351[12])
- H3K27me1 and H3K27me2 (GSE127117: GSM3625691 and GSM3625689, input GSM3625706[13])
- H3K36me2 (GSE126864: SRR8601997, SRR86019978, SRR86019979, inputs SRR8602003, SRR8602004, SRR8602005[14])
- H3K36me3 (ENCODE: GSM6373350 and GSM6373351, inputs GSM4051038, GSM4051039)

Data analysis: All data analyses was performed using R (version 34.3) and Bioconductor (version 3.14). Peak overlap analysis was performed with the package ChIPpeakAnno (version 3.4.2) R package, peaks were annotated using a modified annotatePeak function in the ChIPseeker (version 1.26.2) R package with default parameters and a txdb object created from the GENCODE annotations. Association of gained or lost peaks in MCM2-2A vs WT with peak annotations was tested with Fisher's exact test. The package csaw was used to compute ChIP signal over genome-wide bins of sizes 5KB for broad marks (H3K27me3 and H3K9me3) and 2.5KB was used for narrow marks (H3K4me3, H3K27ac, SUZ12). Bins that weren't log2(1.5) over input in at least one clone were filtered out. Differential occupancy (DO) of bins was performed with edgeR using quasi-likelihood test and bins were deemed significant if FDR < 0.1. DO of ChIP-seq at the promoter levels. To compute ChIP signal over annotated gene promoters, for each TSSs surrounding region counts were obtained using the regionCounts function of the csaw package. Different region sizes were tested (+/-1kb, 2kb, 3kb, 5kb and 10kb) and for each PTM the best size was selected based on the highest correlation with gene expression (see below). Differential binding at TSSs was performed with edgeR (version 3.32.1) using quasi-likelihood test and promoters were deemed significant with cutoff FDR < 0.01. ChIP-seq signal over gene levels was obtained using the overlapResults function of csaw. A combined p-value and log2FC was obtained using the results from the DO TSSs analyses. After correlating gene-level ChIP-seq with RNA-seq log2FC, we found the optimal regions size surrounding the TSSs to be: H3K27ac = +/-10KB, H3K27me3 = +/-1kb, H3K9me3 = +/-5kb, H3K4me3 = +/-1kb and SUZ12 = +/-1kb.

ChIP signal over repeat subfamilies was quantified with the TEcount program (see RNA-seq methods) on the STAR aligned bam files. Differential ChIP signal between MCM2-2A and WT samples was computed using DESeq2 (version 1.30.1) , log2 Fold changes were shrunk

using the apeglm method[15] for visualization in order to give less weight to subfamilies with low counts. Global increase of histone mark levels (RRPMs) in MCM2-2A clones vs WT clones was tested with an upper-tail Wilcoxon signed rank test.

Promoter chromatin state analysis: Chromatin states were defined for promoters, defined by annotated TSSs (GENCODE vM23), whose genes were supported by RNA-seq expression data in any of the considered clones. The presence of ChIP-seq peaks of H3K4me3, H3K27me3, H3K9me3 and H3K27ac called in WT#1 within 1kb of promoters were considered. Association of gene DE status with WT chromatin state was tested using Fisher's exact test comparing the frequency of a state in DE promoters versus all other expressed promoters.

RNA-seq

Data processing: Sequences were trimmed for adapters and filtering for low quality reads was performed with trimmomatic (version 0.39) with default parameters. Mapping (mm10) was performed with STAR (version 2.7.1a) with parameters –twopassMode Basic –twopass1readsN -1 –alignSJDBoverhangMin 10 and multimapping parameters -winAnchorMultimapNmax 200 –outFilterMultimapNmax 100 for further repeats quantification as suggested in[16].

Transcribed repetitive elements: Quantification of repeat subfamilies was performed on the processed bam files with the program Tecount (version 2.1.3), part of the Tetranscripts package[17], using the provided curated repeat annotations for mm10 (http://labshare.cshl.edu/shares/mhammelllab/www-data/TEtranscripts/TE_GTF/). To obtain

count values at the loci level, the Telocal (version 1.1.1) program (also part the Tetoolkit suit of tools) was used, using the same parameters as Tecount.

Data analysis: Differential expression analysis between MCM2-2A and WT clones was performed with Deseq2 accounting for batch effects detected in PCA analysis, product of different sequencing runs. Differentially expressed (DE) genes/repeat subfamilies were defined as those with |FC| > 1.5 and FDR < 0.01. Heatmaps were created with the pheatmap R package, GO term functional enrichment was performed with the package clusterProfiler[18] (version 3.18.1). Gene Set Enrichment Analysis (GSEA)[19] was preformed using GSEA software (version 4.0.3) to test the enrichment of gene sets specifically expressed in early embryonic or 2C-like cells [20-22] in MCM2-2A DE genes. Raw fastq files for SUZ12-KO[23] (GSE127804), SETDB1-KO[24] (BioProject PRJNA544540) and SUV39H1/2-dKO[25] (GSE57092) experiments were downloaded and processed in the same way as MCM2-2A RNA-seq to obtain lists of DE genes and repeats used for overlap analyses.

scRNA-seq

Data processing: Data processing: 3' cell multiplexing data was converted with Cell Ranger (version 6.0.2) to acquire raw reads (cellranger mkfastq) and subsequently sparse matrices (cellranger multi) based on GENCODE vM23 transcriptome annotations. To identify cell doublets and outliers all samples were first log-normalized and scaled individually via Seurat (version 4.0.4). Integration of all samples was based on identifying anchors by mutual nearest neighbours after concerted dimensionality reduction across datasets via diagonalized canonical correlation analysis (CCA[26]). Cells corresponding to doublets were identified with DoubletFinder (version 2.0.3) with the doublet formation rate retrieved from Cell Ranger's estimates for homotypic doublets. Cells were defined as outliers when >10% of a cell's total

number of reads were mapped to mitochondrial genes. To perform RNA velocity analyses, the 3' cell multiplexing data was processed with velocyto (version 0.17.17) based on read alignments derived from Cell Ranger to acquire sparse matrices for both spliced and unspliced transcripts per sample. The resulting loom files were read with SeuratWrappers (version 0.3.0) and the velocyto-derived Seurat objects were filtered against cells identified as doublets or outliers. Standard processing for all samples included log-normalization and variable feature detection before and scaling, dimensionality reduction (i.e., PCA, UMAP) and cluster identification before and after CCA-based sample integration. Clustree (version 0.4.3) was used to optimise the resolution of detected clusters.

Data analysis: Integrated Seurat objects were converted via SeuratWrappers into cell data set objects amenable for monocle3 (version 1.0.0) to learn pseudotime trajectories separately for WT, MCM2 and MCM2-R samples. Transcriptome velocities were acquired based on SeuratWrappers, velocyto.R (version 0.6) and the velocyto-derived transcript counts. Custom R objects and functions were devised to visualize velocities on UMAP embeddings via ggplot2 (version 3.3.5). Cells were further assigned cell cycle phases via the cyclone function in scran (version 1.18.7) based on mouse cell cycle markers provided by that package. All expression values for individual genes per cell in the UMAP embedding and in the density plots were Seurat-derived log-normalized counts after CCA-integration for spliced transcripts. Quantification of transcribed repetitive elements was obtained using the scTE program (version 1.0)[27].

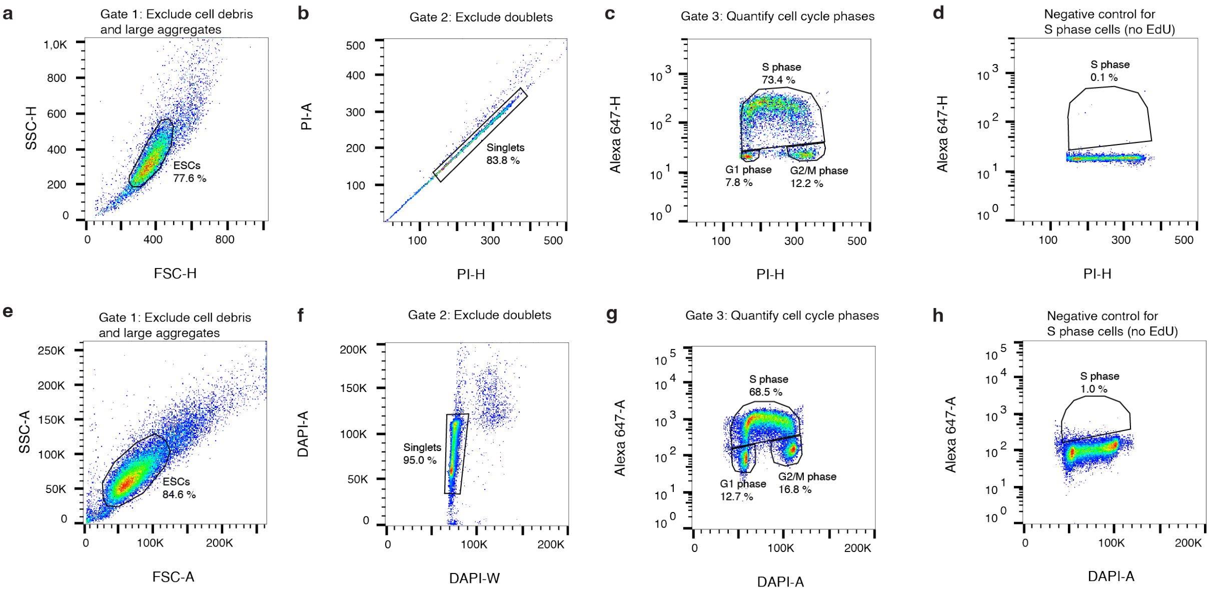

Supplementary Fig. 1. Flow cytometry gating strategy for cell cycle analysis. Representative example of gating strategy for experiments with MCM2-2A ESCs (a-d) shown in Extended Data Fig. 1d-f, and for experiments with POLE4 KO ESCs (e-h) shown in Extended Data Fig.8b. For MCM2-2A experiments, cells were stained with Propidium Iodide (PI) to visualize DNA content and analyzed on a FACS Calibur instrument. For POLE4 KO experiments, cells were stained with DAPI to visualize DNA content and analyzed on a LSR Fortessa instrument. Note that the overall gating strategy was the same for all experiments: The main cell population was selected by excluding cell debris and large aggregates (a, e), followed by selection of singlets (b, f). The cell cycle distribution was then analysed based on EdU incorporation and DNA content (c, g), where a “no EdU”-sample was used as negative control for S phase cells (d, h).

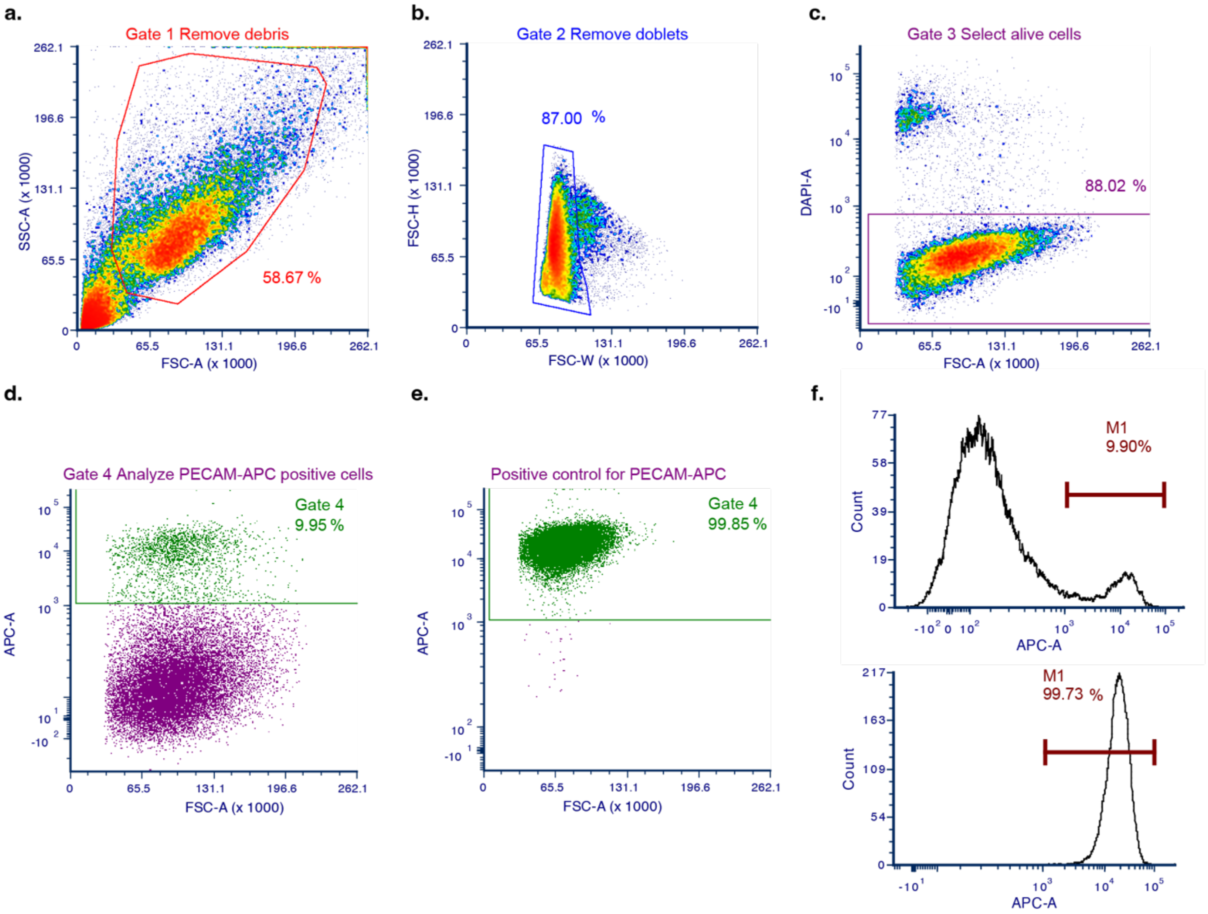

Supplementary Fig. 2. Flow cytometry gating strategy for neuronal differentiation.
Representative example of gating strategy for PECAM positive cells during neuronal differentiation shown in Fig. 6b and Extended Data Fig. 10a. The main cell population was selected by excluding cell debris and large aggregates (a), followed by selection of singlets (b), followed by a selection of live cells or DAPI negative cells (Cells were stained 1:10000 for DAPI) (c). Followed by the analysis of PECAM positive populations. Cells were stained 1:200 for PECAM-APC. Negative and positive controls (e) were used to select the proper PECAM gating. In (f) examples of two histograms showing one clone having only 10% PECAM positive cells and another clone having 100% PECAM positive cells.

Supplementary Table 1. Sequencing data generated in this study.

The file contains information about all SCAR-seq, ChIP-seq, RNA-seq and single-cell RNA-seq samples, which were generated in this study. Datasets are grouped according to experiment.

Column description:

1. Sample ID (identical to GEO sample ID)
2. Experiment ID
3. EdU pulse length (min)
4. Cell line name (internal ID in Groth lab)
5. Clone name (cell line ID used in this study)
6. Replicate name
7. Number of mapped reads
8. Fraction of reads mapped
9. Fraction of duplicated reads (before quality filtering)
10. Number of mapped reads after quality filtering and de-duplication
11. Non-redundant fraction (NRF) for library complexity QC
12. Normalized strand cross-correlation coefficients (NSC) for enrichment QC
13. Relative strand cross-correlation coefficients (RSC) for enrichment QC
14. Number of detected cells
15. Number of filtered cells
16. Average number of spliced reads per filtered cell
17. Average number of spliced features per filtered cell
18. Mode of sequencing

Supplementary Table 2. Antibodies used in this study.

Name	Supplier	Catalog number	Assay	Concentration
H3K27me3	Cell signaling	9733S	ChIP	10 μL/10 μg chromatin for native ChIP<br>5 μL/10 μg chromatin for crosslinked ChIP
H3K4me3	Cell signaling	9751S	ChIP	10 μL/10 μg chromatin
H3K9me3	abcam	ab176916	ChIP	1 μg/10 μg chromatin
H3K27ac	Epicypher	13-0045	ChIP	2.5 μg/10 μg chromatin
H4K20me0	abcam	ab227804	ChIP	1 μg/10 μg chromatin
SUZ12	Cell signaling	3737S	ChIP	2.5-5 μL/10 μg chromatin
Cytokeratin7	Santa Cruz	sc70936	IF	1:200
Gata6 XP	Cell signaling	5851	IF	1:200
Nanog	eBioscience	14-5761	IF	1:200 on cells<br>1:50 on embryos
Otx2	R&D	AF1979	IF	1:150
Pecam APC conj (CD31)	BD Pharmingen	551262	Flow cytometry	1:400
Tuj1	Covance	mms-435p	IF	1:500
Tubulin	Abcam	ab6160	Western Blot	1:10000
POLE4	Gift from S. Boulton[28]	N/A	Western Blot	1:1000

Supplementary Table 3. TALEN and oligonucleotide sequences related to genome editing.

Name	Experiment	Sequence
TALEN-EED	MCM2-2A generation	HD NG HD NG NG NN HD NI NN NI NN NI HD NG NI HD HD NN NG (targets: CTCTTGCAGAGACTACCGT)
TALEN-KKR	MCM2-2A generation	HD HD HD NG HD NN NN HD HD NG HD NN NG NI NN NI HD NI NG (targets: CCCTCGGCCTCGTAGACAT)
sgRNA #1	MCM2-R generation	GCGACATCGAGCTCCGGAAT
sgRNA #2	POLE4-KO generation	CACGTTTCGGGAGGGGATGG
sgRNA #3	POLE4-KO generation	CTCTACCCAAATCTCTCCTC
Oligonucleotide donor #1*	MCM2-2A generation	TTTGGGGATTCATTGTCCACTGTTGGTCTCTTGCAGAGACGCCCGTCCCATTCCGGAGCTCGATGTCGCCGAGGCCGAGGGATTGGCCCTGGATGATGAAGATGTGGAG
Oligonucleotide donor #2†	MCM2-R generation	TTTGGGGATTCATTGTCCACTGTTGGTCTCTTGCAGAGACTACCGTCCCATTCCTGAGCTCGATGTCTACGAGGCCGAGGGATTGGCCCTGGATGATGAAGATGTGGAG
Primer #1 (forward)	MCM2-2A and MCM2-R genotyping	ATCTAGAGGAAGCACTGGCCAC
Primer #2 (reverse)	MCM2-2A and MCM2-R genotyping	GAAGTTCTTGAAGCGGTGGTGG
Primer #3 (forward, for sequencing)	MCM2-2A and MCM2-R genotyping	CAGCAAGGACTTTGTAAGCCCG
Primer #4 (forward)	POLE4-KO genotyping	AAGGGGCCGAAATCGCG
Primer #5 (reverse)	POLE4-KO genotyping	TCCCCTTGCTTCAATGATGCC
Primer #6 (reverse, in deleted region)	POLE4-KO genotyping	GCAATCCTGTGTAGACGTGGAC

* 4 point mutations used to introduce Y81A and Y90A are underlined. Introducing the Y90A mutations disrupted the AccI restriction site, which was used as readout for genotyping.

† 5 point mutations are underlined: 4 missense mutations to reverse A81 and A90 back to Y81 and Y90, 1 silent mutation to disrupt gRNA binding site.

Supplementary References

1 Flemr, M. & Buhler, M. Single-Step Generation of Conditional Knockout Mouse Embryonic Stem Cells. *Cell Rep* **12**, 709-716, doi:10.1016/j.celrep.2015.06.051 (2015).

2 Cermak, T. *et al.* Efficient design and assembly of custom TALEN and other TAL effector-based constructs for DNA targeting. *Nucleic Acids Res* **39**, e82, doi:10.1093/nar/gkr218 (2011).

3 MacLean, B. *et al.* Skyline: an open source document editor for creating and analyzing targeted proteomics experiments. *Bioinformatics* **26**, 966-968, doi:10.1093/bioinformatics/btq054 (2010).

4 Petryk, N. *et al.* Genome-wide and sister chromatid-resolved profiling of protein occupancy in replicated chromatin with ChOR-seq and SCAR-seq. *Nat Protoc* **16**, 4446-4493, doi:10.1038/s41596-021-00585-3 (2021).

5 Petryk, N. *et al.* MCM2 promotes symmetric inheritance of modified histones during DNA replication. *Science* **361**, 1389-1392, doi:10.1126/science.aau0294 (2018).

6 Lun, A. T. & Smyth, G. K. csaw: a Bioconductor package for differential binding analysis of ChIP-seq data using sliding windows. *Nucleic Acids Res* **44**, e45, doi:10.1093/nar/gkv1191 (2016).

7 Hiratani, I. *et al.* Global reorganization of replication domains during embryonic stem cell differentiation. *PLoS Biol* **6**, e245, doi:10.1371/journal.pbio.0060245 (2008).

8 Orlando, D. A. *et al.* Quantitative ChIP-Seq normalization reveals global modulation of the epigenome. *Cell Rep* **9**, 1163-1170, doi:10.1016/j.celrep.2014.10.018 (2014).

9 Zhang, Y. *et al.* Model-based analysis of ChIP-Seq (MACS). *Genome Biol* **9**, R137, doi:10.1186/gb-2008-9-9-r137 (2008).

10 Chen, K. *et al.* Broad H3K4me3 is associated with increased transcription elongation and enhancer activity at tumor-suppressor genes. *Nat Genet* **47**, 1149-1157, doi:10.1038/ng.3385 (2015).

11 Li, Q., Brown, J. B., Huang, H. & Bickel, P. J. Measuring reproducibility of high-throughput experiments. *The Annals of Applied Statistics* **5**, 1752-1779, 1728 (2011).

12 Blackledge, N. P. *et al.* PRC1 Catalytic Activity Is Central to Polycomb System Function. *Mol Cell* **77**, 857-874 e859, doi:10.1016/j.molcel.2019.12.001 (2020).

13 Healy, E. *et al.* PRC2.1 and PRC2.2 Synergize to Coordinate H3K27 Trimethylation. *Mol Cell* **76**, 437-452 e436, doi:10.1016/j.molcel.2019.08.012 (2019).

14 Turberfield, A. H. *et al.* KDM2 proteins constrain transcription from CpG island gene promoters independently of their histone demethylase activity. *Nucleic Acids Res* **47**, 9005-9023, doi:10.1093/nar/gkz607 (2019).

15 Zhu, A., Ibrahim, J. G. & Love, M. I. Heavy-tailed prior distributions for sequence count data: removing the noise and preserving large differences. *Bioinformatics* **35**, 2084-2092, doi:10.1093/bioinformatics/bty895 (2019).

16 Jin, Y. & Hammell, M. Analysis of RNA-Seq Data Using TEtranscripts. *Methods Mol Biol* **1751**, 153-167, doi:10.1007/978-1-4939-7710-9_11 (2018).

17 Jin, Y., Tam, O. H., Paniagua, E. & Hammell, M. TEtranscripts: a package for including transposable elements in differential expression analysis of RNA-seq datasets. *Bioinformatics* **31**, 3593-3599, doi:10.1093/bioinformatics/btv422 (2015).

18 Yu, G., Wang, L. G., Han, Y. & He, Q. Y. clusterProfiler: an R package for comparing biological themes among gene clusters. *OMICS* **16**, 284-287, doi:10.1089/omi.2011.0118 (2012).

19 Subramanian, A. *et al.* Gene set enrichment analysis: a knowledge-based approach for interpreting genome-wide expression profiles. *Proc Natl Acad Sci U S A* **102**, 15545-15550, doi:10.1073/pnas.0506580102 (2005).

20 Deng, Q., Ramskold, D., Reinius, B. & Sandberg, R. Single-cell RNA-seq reveals dynamic, random monoallelic gene expression in mammalian cells. *Science* **343**, 193-196, doi:10.1126/science.1245316 (2014).

21 Eckersley-Maslin, M. A. *et al.* MERVL/Zscan4 Network Activation Results in Transient Genome-wide DNA Demethylation of mESCs. *Cell Rep* **17**, 179-192, doi:10.1016/j.celrep.2016.08.087 (2016).

22 Macfarlan, T. S. *et al.* Embryonic stem cell potency fluctuates with endogenous retrovirus activity. *Nature* **487**, 57-63, doi:10.1038/nature11244 (2012).

23 Hojfeldt, J. W. *et al.* Accurate H3K27 methylation can be established de novo by SUZ12-directed PRC2. *Nat Struct Mol Biol* **25**, 225-232, doi:10.1038/s41594-018-0036-6 (2018).

24 Wu, K. *et al.* SETDB1-Mediated Cell Fate Transition between 2C-Like and Pluripotent States. *Cell Rep* **30**, 25-36 e26, doi:10.1016/j.celrep.2019.12.010 (2020).

25 Bulut-Karslioglu, A. *et al.* Suv39h-dependent H3K9me3 marks intact retrotransposons and silences LINE elements in mouse embryonic stem cells. *Mol Cell* **55**, 277-290, doi:10.1016/j.molcel.2014.05.029 (2014).

26 Stuart, T. *et al.* Comprehensive Integration of Single-Cell Data. *Cell* **177**, 1888-1902 e1821, doi:10.1016/j.cell.2019.05.031 (2019).

27 He, J. *et al.* Identifying transposable element expression dynamics and heterogeneity during development at the single-cell level with a processing pipeline scTE. *Nat Commun* **12**, 1456, doi:10.1038/s41467-021-21808-x (2021).

28 Bellelli, R. *et al.* Polepsilon Instability Drives Replication Stress, Abnormal Development, and Tumorigenesis. *Mol Cell* **70**, 707-721 e707, doi:10.1016/j.molcel.2018.04.008 (2018).
