# Supplementary material for: Symmetric inheritance of parental histones governs epigenome maintenance and embryonic stem cell identity
Source: Nat Genet. 2023 Sep 4;55(9):1567–78. doi: 10.1038/s41588-023-01476-x (PMC10484787; doi:10.1038/s41588-023-01476-x)
Supplement: Supplementary file 2 — Reporting Summary [file 41588_2023_1476_MOESM2_ESM.pdf]

nature portfolio

Corresponding author(s): Anja Groth, Joshua M. Brickman, Robin Andersson

Last updated by author(s): Jun 13, 2023

Reporting Summary

Nature Portfolio wishes to improve the reproducibility of the work that we publish. This form provides structure for consistency and transparency in reporting. For further information on Nature Portfolio policies, see our Editorial Policies and the Editorial Policy Checklist.

Please do not complete any field with "not applicable" or n/a. Refer to the help text for what text to use if an item is not relevant to your study.
For final submission: please carefully check your responses for accuracy; you will not be able to make changes later.

Statistics

For all statistical analyses, confirm that the following items are present in the figure legend, table legend, main text, or Methods section.

n/a	Confirmed	
☐	☒	The exact sample size (*n*) for each experimental group/condition, given as a discrete number and unit of measurement
☐	☒	A statement on whether measurements were taken from distinct samples or whether the same sample was measured repeatedly
☐	☒	The statistical test(s) used AND whether they are one- or two-sided<br>*Only common tests should be described solely by name; describe more complex techniques in the Methods section.*
☒	☐	A description of all covariates tested
☐	☒	A description of any assumptions or corrections, such as tests of normality and adjustment for multiple comparisons
☐	☒	A full description of the statistical parameters including central tendency (e.g. means) or other basic estimates (e.g. regression coefficient) AND variation (e.g. standard deviation) or associated estimates of uncertainty (e.g. confidence intervals)
☐	☒	For null hypothesis testing, the test statistic (e.g. F, t, r) with confidence intervals, effect sizes, degrees of freedom and P value noted<br>*Give P values as exact values whenever suitable.*
☒	☐	For Bayesian analysis, information on the choice of priors and Markov chain Monte Carlo settings
☒	☐	For hierarchical and complex designs, identification of the appropriate level for tests and full reporting of outcomes
☐	☒	Estimates of effect sizes (e.g. Cohen's d, Pearson's r), indicating how they were calculated

Our web collection on statistics for biologists contains articles on many of the points above.

Software and code

Policy information about availability of computer code

Data collection	-fasterq-dump from sra-toolkit (v. 2.10) was used to obtain all public external raw sequencing datasets.<br>- BD FACSDiva (v. 8)- FACS acquisition on the LSR Fortessa.<br>- LASFX software (v. 3.7.3.23245) - for confocal image acquisition.<br>- NextSeq 1000/2000 Control Software (v. 1.4.1.39716) and NextSeq Control Software (v. 4.0.1.41).
Data analysis	quality control: fastqc (v.0.11.5) and multiqc (v. 1.8), trimmomatic (v. 0.39), cutadapt (v. 2.5);<br>mapping: BWA (v. 0.7.17-r1188) for ChIP-seq and SCAR-seq, STAR (v. 2.7.1a) for RNA-seq, deepTools (v. 3.0), macs2 (v. 2.2.4), danpos (v.2.2.2), DESeq2 (v 1.26), bedtools (v. 2.29), samtools (v. 1.9), TEcount (v. 2.1.3), TElocal (v.1.1.1); Data analyses: R (v. 4.3), bioconductor (v. 3.12), ggplot (v. 3.4.1), ChIP-seq: ChIPseeker (v. 1.26.2), ChIPpeakAnno (v. 3.24.2), edgeR (v. 3.32.1), DESeq2 (v. 1.30.1), csaw (v. 1.24.3), clusterProfiler (v 3.18.1), GSEA (v. 4.0.3). RNA-seq: DESeq2 (v. 1.30.1), clusterProfiler (v 3.18.1), GSEA (v. 4.0.3). scRNA-seq: cellranger (v.6.0.2), Seurat (v. 4.0.4), DoubletFinder (v. 2.0.3), SeuratWrappers (v. 0.3.0), velocyto (version 0.17.17), monocle3 (v. 1.0.0), velocyto.R (v. 0.6), scran (v. 1.18.7), Clustree (v. 0.4.3), scTE (v. 1.0.)<br><br>Scripts and instructions for data processing and analyses are available at: https://github.com/anderssonlab/Wenger_et_al_2023<br><br>FlowJo (v.10.4.2)<br>FCS Express 6.0 (v. 6.0).<br>GraphPad Prism (v. 9)

For manuscripts utilizing custom algorithms or software that are central to the research but not yet described in published literature, software must be made available to editors and reviewers. We strongly encourage code deposition in a community repository (e.g. GitHub). See the Nature Portfolio guidelines for submitting code & software for further information.

Data

Policy information about availability of data

All manuscripts must include a data availability statement. This statement should provide the following information, where applicable:

- Accession codes, unique identifiers, or web links for publicly available datasets
- A description of any restrictions on data availability
- For clinical datasets or third party data, please ensure that the statement adheres to our policy

Sequence data generated in this study have been deposited to NCBI GEO with the accession code GSE154391 (https://www.ncbi.nlm.nih.gov/geo/query/acc.cgi?acc=GSE154391). Proteomics data that support the findings of this study have been deposited to ProteomeXchange via PRIDE with the accession codes PXD020326 and PXD030364.
We used the following data to analyze our genomic data:
We used as reference genome Mus musculus mm10 and the Drosophila melanogaster dm6.
for genome annotations we used GENCODE vM23 (https://ftp.ebi.ac.uk/pub/databases/gencode/Gencode_mouse/release_M23/gencode.vM23.annotation.gtf.gz).
ENCODE Black list (https://storage.googleapis.com/encode-pipeline-genome-data/mm10/mm10.blacklist.bed.gz).
Repeat subfamily annotations (https://labshare.cshl.edu/shares/mhammelllab/www-data/TEtranscripts/TE_GTF/GRCm38_GENCODE_rmsk_TE.gtf.gz)
mESC OK-seq initiation zones (https://ftp.ncbi.nlm.nih.gov/geo/samples/GSM3290nnn/GSM3290342/suppl/GSM3290342_Ok_IZ.txt.gz)
We re-analyzed the following mESC ChIP and RNA sequencing data:
H2AK119ub1 (GSE132752: GSM3891343 and GSM3891344, inputs GSM3891350, GSM3891351); H3K27me1 and H3K27me2 (GSE127117: GSM3625691 and GSM3625689, input GSM3625706); H3K36me2 (GSE126864: SRR8601997, SRR86019978, SRR86019979, inputs SRR8602003, SRR8602004, SRR8602005); H3K36me3 (ENCODE: GSM6373350 and GSM6373351, inputs GSM4051038, GSM4051039); SUZ12-KO RNA-seq (GSE127804); SETDB1-KO RNA-seq (BioProject PRJNA544540) and SUV39H1/2-dKO RNA-seq (GSE57092).

Field-specific reporting

Please select the one below that is the best fit for your research. If you are not sure, read the appropriate sections before making your selection.

- [x] Life sciences
- [ ] Behavioural & social sciences
- [ ] Ecological, evolutionary & environmental sciences

Life sciences study design

All studies must disclose on these points even when the disclosure is negative.

Sample size	No statistical method was used to predetermine sample size. ChIP/SCAR-seq experiments were performed in a minimum of 2 biological replicates to assure reproducibility according to the ENCODE ChIP guidelines (https://www.encodeproject.org/chip-seq/histone-encode4/). For RNA-seq we performed a minimum of 3 biological replicates or 2 biological replicates when comparing multiple mutant clones where the clones serve as biological replicates following the ENCODE RNA-seq guidelines (https://www.encodeproject.org/data-standards/encode4-bulk-rna/#standards). All other experiments involving quantification were performed in a minimum of 3 biological replicates according to the standard of the field.
Data exclusions	No samples were excluded from analysis.
Replication	All results were tested and confirmed with at least two independent biological experiments including 2 or more independent MCM2-2A, POLE4-KO, or MCM2-R ESC clones, to assure reproducibility of the results. The single-cell RNA-seq experiment was performed in thousands of cells in WT, MCM2-2A clone and MCM2-R clone. Note that replicate information for each experiment is provided in the figure legends and in a detailed list of all sequencing data generated in this study provided in Supplementary Table 1.
Randomization	No method of randomization was applied as the study does not involve a clinical trial or human subjects. All cell lines, WT, mutant and rescue cells were cultured together to reduce variability which is not the result of treatment/mutation.
Blinding	No blinding method was applied. Genomic, proteomic and cell cytometry analysis does not apply subjective measurement. Chimera analyses were performed and scored by two different individuals.

Reporting for specific materials, systems and methods

We require information from authors about some types of materials, experimental systems and methods used in many studies. Here, indicate whether each material, system or method listed is relevant to your study. If you are not sure if a list item applies to your research, read the appropriate section before selecting a response.

Materials & experimental systems

n/a	Involved in the study	
	X	Antibodies
	X	Eukaryotic cell lines
X		Palaeontology and archaeology
	X	Animals and other organisms
X		Human research participants
X		Clinical data
X		Dual use research of concern

Methods

n/a	Involved in the study	
	X	ChIP-seq
	X	Flow cytometry
X		MRI-based neuroimaging

Antibodies

Antibodies used

Target_Supplier name_Catalog number_Clone name_Lot number_Application
H3K27me3_Cell Signaling_9733S_C36B11_14 and 16_ChIP
H3K4me3_Cell Signaling_9751S_C42D8_10_ChIP
H3K9me3_abcam_ab176916_EPR16601_GR3218257-2_ChIP
H3K27ac_Epicypher_13-0045_n/a_20120001-28_ChIP
H4K20me0_abcam_ab227804_EPR22116_GR3269933-7_ChIP
SUZ12_Cell Signaling_3737S_D39F6_8_ChIP
Cytokeratin7_Santa Cruz_sc70936_5F282_LO210_IF
Gata6 XP_Cell Signaling_5851_D61E4_2_IF
Nanog_eBioscience_14-5761_eBioMLC-51_IF
Otx2_R&D_AF1979_n/a_KNO0921031_IF
Pecam APC conj. (CD31)_BD Pharmingen_551262_MEC 13.3_6133900_Flow Cytometry
Tuj1_Covance_mms-435p-250_TUJ1_d13AF00117_IF
Tubulin_abcam_ab6160_YL1/2_n/a_Western Blot
POLE4_gift from S. Boulton_n/a_n/a_n/a_Western Blot

Validation

All commercial antibodies were validated by the suppliers. The antibody against POLE4 was home-made and validated by the Boulton lab (Bellelli et al, Mol Cell 2018). Additional information regarding specificity of the histone PTM antibodies used for ChIP is provided below.

H3K27me3: The manufacturer has validated this antibody for ChIP using SimpleChIP Enzymatic Chromatin IP Kits and has determined the specificity by peptide ELISA. The manufacturer states: "The antibody does not cross-react with non-methylated, mono-methylated or di-methylated Lys27. In addition, the antibody does not cross-react with mono-methylated, di-methylated or tri-methylated histone H3 at Lys4, Lys9, Lys36 or Histone H4 at Lys20." In addition, this antibody is widely used and has >800 product citations.

H3K4me3: The manufacturer has validated this antibody for ChIP using SimpleChIP Enzymatic Chromatin IP Kits and has determined the specificity by Western Blotting (use of competitor peptides). The manufacturer states: "This antibody shows some cross-reactivity with histone H3 that is di-methylated on Lys4, but does not cross-react with non-methylated or mono-methylated histone H3 Lys4. In addition, the antibody does not cross-react with methylated histone H3 Lys9, Lys27, Lys36 or methylated histone H4 Lys20." In addition, this antibody is widely used and has >350 product citations.

H3K9me3: The manufacturer has validated this antibody for ChIP and has determined the specificity in a peptide array against 501 histone peptides (moderate cross-reactivity with 2 peptides: H3K4cr and H2BK20cr). In addition, this antibody was tested by Epicypher in a SNAP-ChIP assay (spike-in of barcoded recombinant nucleosomes) and passed both the specificity and IP efficiency criteria (personal communication with Danielle Maryanski).

H3K27ac: This antibody was validated by the manufacturer and meets the "SNAP-ChIP® Certified" criteria for specificity and efficient target enrichment in a ChIP experiment (<20% cross-reactivity across the panel, >5% recovery of target input). The manufacturer states: "This antibody reacts to H3K27ac and no cross reactivity with other lysine acylations in the EpiCypher SNAP-ChIP K-AcylStat panel, is detected. Antibody binding to H3K27ac in the context of phosphorylation at S28 (H3K27acS28ph) is inhibited to varying degrees in Luminex and ChIP."

H4K20me0: The manufacturer has validated this antibody for ChIP and has determined the specificity in a peptide array (moderate cross-reactivity with R19me1, R19me2a and R23me1).

SUZ12: The manufacturer has validated this antibody for ChIP using SimpleChIP Enzymatic Chromatin IP Kits and specify that the antibody can detects endogenous levels of SUZ12 protein.

Cytokeratin7: Recommended my the manufacturer for detection of Cytokeratin 7 of mouse, rat, human, hamster, canine and porcine origin by WB, IP, IF, IHC(P) and FCM.

Gata6: The manufacturer has validated this antibody for ChIP using SimpleChIP Enzymatic Chromatin IP Kits and is certified for the usage for cut&run, ChIP-seq, Flow cytometry, Immunofluorescence (IF) and Western Blot (WB) for detection of endogenous protein levels.

Nanog: The manufacturer validated the specificity of the antibody using both relative expression comparing the Nanog protein levels using western blot in F9 cell line (cell line that is known to express NANOG) to other cell line which are know to be negative to Nanog.

The antibody was shown in multiple published research for IF and WB.

PECAM APC conj. (CD31): The manufacturer routinely test the antibody for its application in flow cytometry.

Tuj1: The antibody is verified by the manufacturer for WB and ICC and each lot is quality control tested by formalin-fixed paraffin embedded immunohistochemical staining.

Tubulin: The antibody is validated as Western Blot loading control by the manufacturer and its performance is guaranteed for Flow Cytometry, IF and IHC-P.

Eukaryotic cell lines

Policy information about cell lines

Cell line source(s)	E14JU cell line with a 129/Ola background was derived at the Brickman lab (Hamilton et al. 2013)
Authentication	The used cell line was not authenticated.
Mycoplasma contamination	All used cell lines were tested regularly for mycoplasma contamination and tested negative.
Commonly misidentified lines (See ICLAC register)	No commonly misidentified cell lines were used in this study.

Animals and other organisms

Policy information about studies involving animals; ARRIVE guidelines recommended for reporting animal research

Laboratory animals	8 C57BL/6NRj female mice a (4 weeks) underwent super-ovulation to obtain morulae. Embryos background C57BL/6NRj were injected to 32 CD1 (RjOrk:SWISS) receptor female (9-13 weeks).
Wild animals	This study did not involve wild animals.
Field-collected samples	This study did not involve samples collected from the field.
Ethics oversight	All animal work was carried in accordance with European legislation. All work was authorized by and carried out under Project License 2018-15-0201-01520 issued by the Danish Regulatory Authority.

Note that full information on the approval of the study protocol must also be provided in the manuscript.

ChIP-seq

Data deposition

☒ Confirm that both raw and final processed data have been deposited in a public database such as GEO.

☒ Confirm that you have deposited or provided access to graph files (e.g. BED files) for the called peaks.

Data access links<br>*May remain private before publication.*	Sequencing data have been deposited to NCBI GEO under accession number GSE154391. Access: https://www.ncbi.nlm.nih.gov/geo/query/acc.cgi?acc=GSE154391; token: mfyjiwkudrkrruj. All ChIP-seq data are collected in the sub-series GSE154379.
Files in database submission	ChIP_410_H3K27me3_r1<br>ChIP_421_H3K27me3_r1<br>ChIP_439_H3K27me3_r1<br>ChIP_442_H3K27me3_r1<br>ChIP_410_H3K27me3_r2<br>ChIP_421_H3K27me3_r2<br>ChIP_439_H3K27me3_r2<br>ChIP_442_H3K27me3_r2<br>ChIP_410_K27me3_input_r1<br>ChIP_421_K27me3_input_r1<br>ChIP_439_K27me3_input_r1<br>ChIP_442_K27me3_input_r1<br>ChIP_410_K27me3_input_r2<br>ChIP_421_K27me3_input_r2<br>ChIP_439_K27me3_input_r2<br>ChIP_442_K27me3_input_r2<br>ChIP_410_H3K4me3_r1<br>ChIP_421_H3K4me3_r1<br>ChIP_439_H3K4me3_r1<br>ChIP_442_H3K4me3_r1<br>ChIP_410_H3K4me3_r2<br>ChIP_421_H3K4me3_r2

ChIP_439_H3K4me3_r2
ChIP_442_H3K4me3_r2
ChIP_410_K4me3_input_r1
ChIP_421_K4me3_input_r1
ChIP_439_K4me3_input_r1
ChIP_442_K4me3_input_r1
ChIP_410_K4me3_input_r2
ChIP_421_K4me3_input_r2
ChIP_439_K4me3_input_r2
ChIP_442_K4me3_input_r2
ChIP_410_H3K9me3_r1
ChIP_421_H3K9me3_r1
ChIP_439_H3K9me3_r1
ChIP_442_H3K9me3_r1
ChIP_410_H3K9me3_r2
ChIP_421_H3K9me3_r2
ChIP_439_H3K9me3_r2
ChIP_442_H3K9me3_r2
ChIP_410_K9me3_input_r1
ChIP_421_K9me3_input_r1
ChIP_439_K9me3_input_r1
ChIP_442_K9me3_input_r1
ChIP_410_K9me3_input_r2
ChIP_421_K9me3_input_r2
ChIP_439_K9me3_input_r2
ChIP_442_K9me3_input_r2
ChIP_410_H3K27ac_r1
ChIP_421_H3K27ac_r1
ChIP_439_H3K27ac_r1
ChIP_442_H3K27ac_r1
ChIP_410_H3K27ac_r2
ChIP_421_H3K27ac_r2
ChIP_439_H3K27ac_r2
ChIP_442_H3K27ac_r2
ChIP_410_K27ac_input_r1
ChIP_421_K27ac_input_r1
ChIP_439_K27ac_input_r1
ChIP_442_K27ac_input_r1
ChIP_410_K27ac_input_r2
ChIP_421_K27ac_input_r2
ChIP_439_K27ac_input_r2
ChIP_442_K27ac_input_r2
ChIP_410_SUZ12_r1
ChIP_439_SUZ12_r1
ChIP_442_SUZ12_r1
ChIP_410_SUZ12_r2
ChIP_439_SUZ12_r2
ChIP_442_SUZ12_r2
ChIP_410_SUZ12_r3
ChIP_439_SUZ12_r3
ChIP_442_SUZ12_r3
ChIP_410_SUZ12_input_r1
ChIP_439_SUZ12_input_r1
ChIP_442_SUZ12_input_r1
ChIP_410_SUZ12_input_r2
ChIP_439_SUZ12_input_r2
ChIP_442_SUZ12_input_r2
ChIP_410_SUZ12_input_r3
ChIP_439_SUZ12_input_r3
ChIP_442_SUZ12_input_r3
ChIP_410_H3K27me3_rescue_exp_r1
ChIP_442_H3K27me3_rescue_exp_r1
ChIP_588_H3K27me3_rescue_exp_r1
ChIP_410_H3K27me3_rescue_exp_r2
ChIP_442_H3K27me3_rescue_exp_r2
ChIP_588_H3K27me3_rescue_exp_r2
ChIP_410_H3K27me3_rescue_exp_r3
ChIP_442_H3K27me3_rescue_exp_r3
ChIP_588_H3K27me3_rescue_exp_r3
ChIP_410_K27me3_input_rescue_exp_r1
ChIP_442_K27me3_input_rescue_exp_r1
ChIP_588_K27me3_input_rescue_exp_r1
ChIP_410_K27me3_input_rescue_exp_r2
ChIP_442_K27me3_input_rescue_exp_r2
ChIP_588_K27me3_input_rescue_exp_r2
ChIP_410_K27me3_input_rescue_exp_r3

ChIP_442_K27me3_input_rescue_exp_r3
ChIP_588_K27me3_input_rescue_exp_r3
ChIP_410_H3K9me3_rescue_exp_r1
ChIP_442_H3K9me3_rescue_exp_r1
ChIP_588_H3K9me3_rescue_exp_r1
ChIP_410_H3K9me3_rescue_exp_r2
ChIP_442_H3K9me3_rescue_exp_r2
ChIP_588_H3K9me3_rescue_exp_r2
ChIP_410_H3K9me3_rescue_exp_r3
ChIP_442_H3K9me3_rescue_exp_r3
ChIP_588_H3K9me3_rescue_exp_r3
ChIP_410_K9me3_input_rescue_exp_r1
ChIP_442_K9me3_input_rescue_exp_r1
ChIP_588_K9me3_input_rescue_exp_r1
ChIP_410_K9me3_input_rescue_exp_r2
ChIP_442_K9me3_input_rescue_exp_r2
ChIP_588_K9me3_input_rescue_exp_r2
ChIP_410_K9me3_input_rescue_exp_r3
ChIP_442_K9me3_input_rescue_exp_r3
ChIP_588_K9me3_input_rescue_exp_r3
ChIP_410_H3K4me3_rescue_exp_r1
ChIP_442_H3K4me3_rescue_exp_r1
ChIP_588_H3K4me3_rescue_exp_r1
ChIP_410_H3K4me3_rescue_exp_r2
ChIP_442_H3K4me3_rescue_exp_r2
ChIP_588_H3K4me3_rescue_exp_r2
ChIP_410_H3K4me3_rescue_exp_r3
ChIP_442_H3K4me3_rescue_exp_r3
ChIP_588_H3K4me3_rescue_exp_r3
ChIP_410_K4me3_input_rescue_exp_r1
ChIP_442_K4me3_input_rescue_exp_r1
ChIP_588_K4me3_input_rescue_exp_r1
ChIP_410_K4me3_input_rescue_exp_r2
ChIP_442_K4me3_input_rescue_exp_r2
ChIP_588_K4me3_input_rescue_exp_r2
ChIP_410_K4me3_input_rescue_exp_r3
ChIP_442_K4me3_input_rescue_exp_r3
ChIP_588_K4me3_input_rescue_exp_r3

Genome browser session
(e.g. UCSC)

http://genome.ucsc.edu/s/nalcaraz/rep_chromatin_TC

Methodology

Replicates

Histone PTM ChIP-seq experiments for H3K27me3, H3K4me3, H3K9me3 and H3K27ac were performed in WT cells and 3 MCM2-2A clones (2 biological replicates). In addition, ChIP-seq for H3K27me3, H3K9me3 and H3K4me3 was performed in the rescue cell line MCM2-R and these experiments included WT cells, 1 MCM2-2A clone and 1 MCM2-R clone (3 biological replicates). The SUZ12 ChIP-seq experiment was performed in WT cells and 2 MCM2-2A clones (3 biological replicates).

Sequencing depth

Experiment Mapped_reads Uniquely_mapped_reads read_length single/paired
ChIP_410_H3K27me3_r1 32491978 25222138 76 single
ChIP_421_H3K27me3_r1 25298114 19634316 76 single
ChIP_439_H3K27me3_r1 33617513 25732354 76 single
ChIP_442_H3K27me3_r1 32988896 25002130 76 single
ChIP_410_H3K27me3_r2 29959330 23393559 76 single
ChIP_421_H3K27me3_r2 33565613 25559998 76 single
ChIP_439_H3K27me3_r2 35667612 27021800 76 single
ChIP_442_H3K27me3_r2 32684412 24495047 76 single
ChIP_410_K27me3_input_r1 7632686 6071150 76 single
ChIP_421_K27me3_input_r1 8776765 6907667 76 single
ChIP_439_K27me3_input_r1 7758642 6089336 76 single
ChIP_442_K27me3_input_r1 7992773 6320693 76 single
ChIP_410_K27me3_input_r2 8609543 6834663 76 single
ChIP_421_K27me3_input_r2 7772332 6121143 76 single
ChIP_439_K27me3_input_r2 8408310 6594216 76 single
ChIP_442_K27me3_input_r2 8296248 6545598 76 single
ChIP_410_H3K4me3_r1 65720352 44896610 76 paired-end
ChIP_421_H3K4me3_r1 57616757 39306570 76 paired-end
ChIP_439_H3K4me3_r1 63393741 43961247 76 paired-end
ChIP_442_H3K4me3_r1 61731372 42881472 76 paired-end
ChIP_410_H3K4me3_r2 62675087 42767767 76 paired-end
ChIP_421_H3K4me3_r2 51793906 35973361 76 paired-end
ChIP_439_H3K4me3_r2 63035662 43717478 76 paired-end
ChIP_442_H3K4me3_r2 61127874 42169818 76 paired-end
ChIP_410_K4me3_input_r1 16443636 12306142 76 paired-end
ChIP_421_K4me3_input_r1 16331611 12196614 76 paired-end

ChIP_439_K4me3_input_r1 19436777 14481312 76 paired-end
ChIP_442_K4me3_input_r1 17805958 13365970 76 paired-end
ChIP_410_K4me3_input_r2 21931549 16473868 76 paired-end
ChIP_421_K4me3_input_r2 18487187 13827884 76 paired-end
ChIP_439_K4me3_input_r2 17249031 12859664 76 paired-end
ChIP_442_K4me3_input_r2 17827227 13364862 76 paired-end
ChIP_410_H3K9me3_r1 30121814 11001514 76 single
ChIP_421_H3K9me3_r1 24924740 14818722 76 single
ChIP_439_H3K9me3_r1 35409588 17846281 76 single
ChIP_442_H3K9me3_r1 38887065 20510186 76 single
ChIP_410_H3K9me3_r2 29906145 10525622 76 single
ChIP_421_H3K9me3_r2 37202731 21728749 76 single
ChIP_439_H3K9me3_r2 31062226 15473030 76 single
ChIP_442_H3K9me3_r2 32940263 17203484 76 single
ChIP_410_K9me3_input_r1 7485223 5962574 76 single
ChIP_421_K9me3_input_r1 8761128 6920836 76 single
ChIP_439_K9me3_input_r1 8281931 6521075 76 single
ChIP_442_K9me3_input_r1 9368183 7410857 76 single
ChIP_410_K9me3_input_r2 8242269 6547378 76 single
ChIP_421_K9me3_input_r2 9031566 7133745 76 single
ChIP_439_K9me3_input_r2 7912067 6226767 76 single
ChIP_442_K9me3_input_r2 8143784 6426369 76 single
ChIP_410_H3K27ac_r1 71159842 57975080 76 paired-end
ChIP_421_H3K27ac_r1 62535635 49602422 76 paired-end
ChIP_439_H3K27ac_r1 74139004 59663534 76 paired-end
ChIP_442_H3K27ac_r1 61668157 49142638 76 paired-end
ChIP_410_H3K27ac_r2 63713987 51796456 76 paired-end
ChIP_421_H3K27ac_r2 85381307 67699946 76 paired-end
ChIP_439_H3K27ac_r2 84929755 68912606 76 paired-end
ChIP_442_H3K27ac_r2 65638553 53573060 76 paired-end
ChIP_410_K27ac_input_r1 20525855 15734274 76 paired-end
ChIP_421_K27ac_input_r1 19642875 14944848 76 paired-end
ChIP_439_K27ac_input_r1 17396774 13190286 76 paired-end
ChIP_442_K27ac_input_r1 21567354 16558462 76 paired-end
ChIP_410_K27ac_input_r2 21567362 16537874 76 paired-end
ChIP_421_K27ac_input_r2 21034645 16168984 76 paired-end
ChIP_439_K27ac_input_r2 20882713 16057536 76 paired-end
ChIP_442_K27ac_input_r2 27184281 21100088 76 paired-end
ChIP_410_SUZ12_r1 56748290 42871748 76 paired-end
ChIP_439_SUZ12_r1 45169429 33960370 76 paired-end
ChIP_442_SUZ12_r1 51210327 38151270 76 paired-end
ChIP_410_SUZ12_r2 62831032 47322686 76 paired-end
ChIP_439_SUZ12_r2 53370258 39799466 76 paired-end
ChIP_442_SUZ12_r2 47826878 35969164 76 paired-end
ChIP_410_SUZ12_r3 57026135 43274542 76 paired-end
ChIP_439_SUZ12_r3 55726908 42144886 76 paired-end
ChIP_442_SUZ12_r3 58359504 44126620 76 paired-end
ChIP_410_SUZ12_input_r1 28819053 20758110 76 paired-end
ChIP_439_SUZ12_input_r1 31285062 22542748 76 paired-end
ChIP_442_SUZ12_input_r1 30316185 22003736 76 paired-end
ChIP_410_SUZ12_input_r2 29613212 21406884 76 paired-end
ChIP_439_SUZ12_input_r2 30142234 21738580 76 paired-end
ChIP_442_SUZ12_input_r2 28212447 20469294 76 paired-end
ChIP_410_SUZ12_input_r3 31125255 22598262 76 paired-end
ChIP_439_SUZ12_input_r3 28465502 20586140 76 paired-end
ChIP_442_SUZ12_input_r3 29531116 21509152 76 paired-end
ChIP_410_H3K27me3_rescue_exp_r1 98957907 78058112 100 paired-end
ChIP_442_H3K27me3_rescue_exp_r1 112493700 84062398 100 paired-end
ChIP_588_H3K27me3_rescue_exp_r1 97095767 76298632 100 paired-end
ChIP_410_H3K27me3_rescue_exp_r2 116346766 88989298 100 paired-end
ChIP_442_H3K27me3_rescue_exp_r2 86310237 64824554 100 paired-end
ChIP_588_H3K27me3_rescue_exp_r2 117080433 91154260 100 paired-end
ChIP_410_H3K27me3_rescue_exp_r3 135722186 104432222 100 paired-end
ChIP_442_H3K27me3_rescue_exp_r3 141351073 104692972 100 paired-end
ChIP_588_H3K27me3_rescue_exp_r3 119275912 93167928 100 paired-end
ChIP_410_K27me3_input_rescue_exp_r1 84488343 63829180 100 paired-end
ChIP_442_K27me3_input_rescue_exp_r1 94885405 70952962 100 paired-end
ChIP_588_K27me3_input_rescue_exp_r1 85536264 64402900 100 paired-end
ChIP_410_K27me3_input_rescue_exp_r2 75632489 56697640 100 paired-end
ChIP_442_K27me3_input_rescue_exp_r2 74723734 55882006 100 paired-end
ChIP_588_K27me3_input_rescue_exp_r2 73253757 55483354 100 paired-end
ChIP_410_K27me3_input_rescue_exp_r3 72402471 54639512 100 paired-end
ChIP_442_K27me3_input_rescue_exp_r3 86695015 65436746 100 paired-end
ChIP_588_K27me3_input_rescue_exp_r3 72981457 55318590 100 paired-end
ChIP_410_H3K9me3_rescue_exp_r1 143114351 58566226 100 paired-end
ChIP_442_H3K9me3_rescue_exp_r1 151075938 86737722 100 paired-end

ChIP_588_H3K9me3_rescue_exp_r1 148458388 62323586 100 paired-end
ChIP_410_H3K9me3_rescue_exp_r2 160448597 62627150 100 paired-end
ChIP_442_H3K9me3_rescue_exp_r2 163661702 92296266 100 paired-end
ChIP_588_H3K9me3_rescue_exp_r2 144915853 58187774 100 paired-end
ChIP_410_H3K9me3_rescue_exp_r3 156647118 60414968 100 paired-end
ChIP_442_H3K9me3_rescue_exp_r3 168219311 94809658 100 paired-end
ChIP_588_H3K9me3_rescue_exp_r3 157915955 60519344 100 paired-end
ChIP_410_K9me3_input_rescue_exp_r1 75601299 56599584 100 paired-end
ChIP_442_K9me3_input_rescue_exp_r1 65665757 50660114 100 paired-end
ChIP_588_K9me3_input_rescue_exp_r1 75747161 57449024 100 paired-end
ChIP_410_K9me3_input_rescue_exp_r2 78647803 59398758 100 paired-end
ChIP_442_K9me3_input_rescue_exp_r2 84683290 63534972 100 paired-end
ChIP_588_K9me3_input_rescue_exp_r2 73895779 56332044 100 paired-end
ChIP_410_K9me3_input_rescue_exp_r3 84200577 63591870 100 paired-end
ChIP_442_K9me3_input_rescue_exp_r3 88060636 65743550 100 paired-end
ChIP_588_K9me3_input_rescue_exp_r3 86631920 65084294 100 paired-end
ChIP_410_H3K4me3_rescue_exp_r1 66462448 47982532 100 paired-end
ChIP_442_H3K4me3_rescue_exp_r1 63167689 45223148 100 paired-end
ChIP_588_H3K4me3_rescue_exp_r1 57296517 40945622 100 paired-end
ChIP_410_H3K4me3_rescue_exp_r2 56825175 41309132 100 paired-end
ChIP_442_H3K4me3_rescue_exp_r2 59127229 43148142 100 paired-end
ChIP_588_H3K4me3_rescue_exp_r2 49993212 37045546 100 paired-end
ChIP_410_H3K4me3_rescue_exp_r3 63655156 46522364 100 paired-end
ChIP_442_H3K4me3_rescue_exp_r3 62283572 44991740 100 paired-end
ChIP_588_H3K4me3_rescue_exp_r3 65526508 47314152 100 paired-end
ChIP_410_K4me3_input_rescue_exp_r1 47418077 35486088 100 paired-end
ChIP_442_K4me3_input_rescue_exp_r1 54992100 40624866 100 paired-end
ChIP_588_K4me3_input_rescue_exp_r1 47157345 35345598 100 paired-end
ChIP_410_K4me3_input_rescue_exp_r2 33647078 25084258 100 paired-end
ChIP_442_K4me3_input_rescue_exp_r2 44859931 33317780 100 paired-end
ChIP_588_K4me3_input_rescue_exp_r2 32612897 24298300 100 paired-end
ChIP_410_K4me3_input_rescue_exp_r3 39378061 29368734 100 paired-end
ChIP_442_K4me3_input_rescue_exp_r3 48246102 35795982 100 paired-end
ChIP_588_K4me3_input_rescue_exp_r3 47260047 34973228 100 paired-end

Antibodies

Target_Supplier name_Catalog number_Clone Name_Lot number
H3K27me3_Cell Signaling_9733S_C36B11_14 and 16
H3K4me3_Cell Signaling_9751S_C42D8_10
H3K9me3_abcam_ab176916_EPR16601_GR3218257-2
H3K27ac_Epicypher_13-0045_n/a_20120001-28
SUZ12_Cell Signaling_3737S_D39F6_8

Peak calling parameters

macs2 parameters --nomodel -p 0.01 (H3K4me3, H3K27ac, SUZ12)
and dregion (danpos2 pacakage) with default parameters (H3K27me3, H3K9me3)

Data quality

ChIP-seq libraries were assessed with NFR, PBC1 and PBC2 scores for library complexity, cross correlation scores NSC, RSC, fingerprint plots and Fraction of reads in Peaks.

Software

For processing and peak calling the ENCODE ChIP-seq pipeline (version 1.3.6) was used. Briefly, adapters and low quality reads were filtered with cutadapt (version 2.5), reads were mapped to a hybrid mouse (mm10) and fly (dm3) genome with bwa, duplicate reads were removed with picard (version 2.20.7), ENCODE mm10 blacklist regions were masked and only reads with mapping quality above 30 were considered for further downstream analyses. For peak calling, macs2 was used for narrow histone marks and SUZ12, whereas dregion (from the danpos2 package) was used for calling peaks in broad histone marks.

Flow Cytometry

Plots

Confirm that:

☒ The axis labels state the marker and fluorochrome used (e.g. CD4-FITC).

☒ The axis scales are clearly visible. Include numbers along axes only for bottom left plot of group (a 'group' is an analysis of identical markers).

☒ All plots are contour plots with outliers or pseudocolor plots.

☒ A numerical value for number of cells or percentage (with statistics) is provided.

Methodology

Sample preparation

We used flow cytometry for cell cycle analysis of ESCs and for quantification of cell populations expressing marker genes before and after ESC differentiation. Details of the experimental procedures are provided in the methods section.

Instrument

Cell cycle analysis: BD Biosciences, BD FACS Calibur and LSR Fortessa; Differentiation experiments: BD Biosciences, LSR Fortessa

Software	Cell cycle data were collected with CellQuest Pro and analysed with FlowJo (v.10.4.2). Differentiation data were collected with FACS Diva and analyzed in FCS Express 6 (v. 6) .
Cell population abundance	For the cell cycle analysis mESC are known to have more than 60% cells in S-phase and 10-20% of cells in G1 and G2/M, comparable to our findings. During differentiation we followed gradual reduction in PECAM positive cells, being able to evaluate the subpopulation abundance change during differentiation.
Gating strategy	Cell cycle analysis: 1) FSC/SSC gates were used to define a homogeneous cell population, 2) H/A gates of propidium iodide (DNA content) was used to exclude doublets, 3) For gating EdU-positive cells, a no-EdU sample was used as negative control. (Supplementary Fig. 1)<br>For differentiation analysis: 1) FSC/SSC gates were used to define a homogeneous cell population, 2) FSC H/FSC W gates were used to exclude doublets, 3) DAPI negative gate was used to select live population, 4) Specific surface marker fluorochrome (Pecam-APC) was used to analyze percentage of undifferentiated cells. (Sapplementary Fig. 2)

☒ Tick this box to confirm that a figure exemplifying the gating strategy is provided in the Supplementary Information.
