# Supplementary figures and images for: Symmetric inheritance of parental histones governs epigenome maintenance and embryonic stem cell identity
Source: Nat Genet. 2023 Sep 4;55(9):1567–78. doi: 10.1038/s41588-023-01476-x (PMC10484787; doi:10.1038/s41588-023-01476-x)

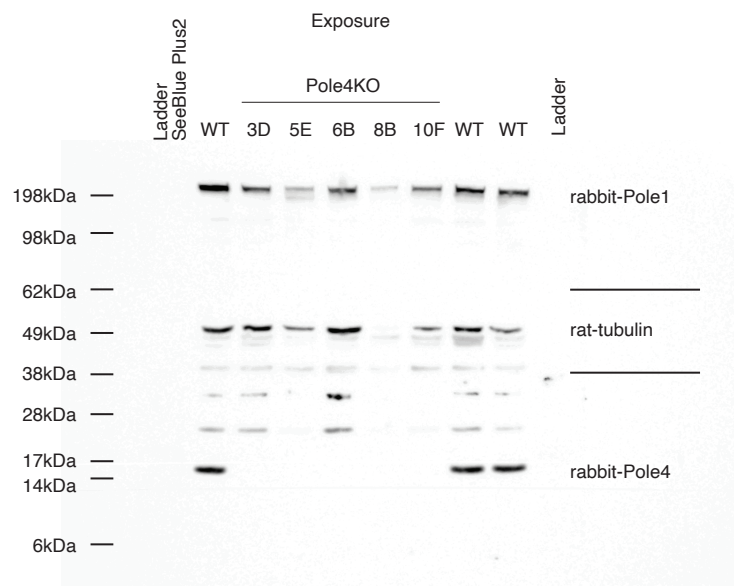

cell line #550: POLE4-KO#3  
 cell line #551: POLE4-KO#1  
 cell line #552: POLE4-KO#2  
 cell line #553: clone 3D  
 cell line #554: remaining clones

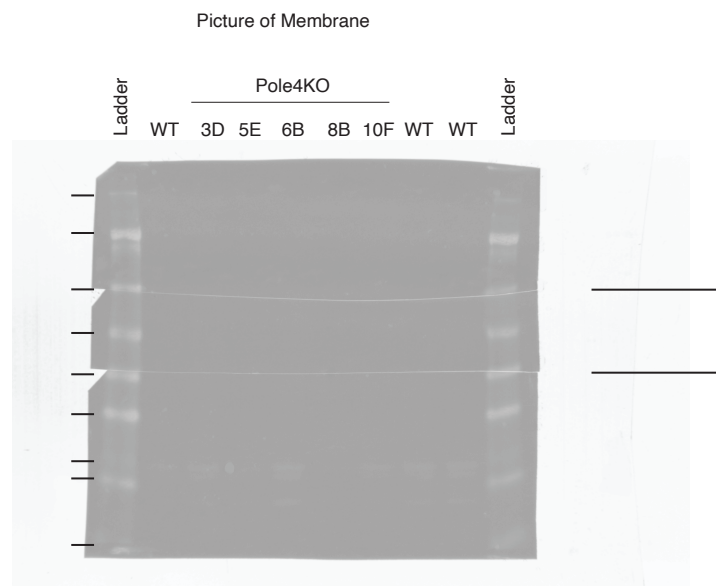

Supplement: Source Data Extended Data Fig. 8 — Unprocessed western blots. [file 41588_2023_1476_MOESM10_ESM.pdf]
